# Risk factors for health impairments in children after hospitalization for acute COVID-19 or MIS-C

**DOI:** 10.3389/fped.2023.1260372

**Published:** 2023-10-18

**Authors:** Aline B. Maddux, Cameron C. Young, Suden Kucukak, Laura D. Zambrano, Margaret M. Newhams, Caitlin K. Rollins, Natasha B. Halasa, Shira J. Gertz, Elizabeth H. Mack, Stephanie Schwartz, Michele Kong, Laura L. Loftis, Katherine Irby, Courtney M. Rowan, Keiko M. Tarquinio, Matt S. Zinter, Hillary Crandall, Natalie Z. Cvijanovich, Jennifer E. Schuster, Julie C. Fitzgerald, Mary A. Staat, Charlotte V. Hobbs, Ryan A. Nofziger, Steven Shein, Heidi Flori, Melissa L. Cullimore, Brandon M. Chatani, Emily R. Levy, Katri V. Typpo, Janet R. Hume, Angela P. Campbell, Adrienne G. Randolph, the Overcoming COVID-19 Investigators, Michele Kong

**Affiliations:** ^1^ Section of Critical Care Medicine, Department of Pediatrics, University of Colorado School of Medicine and Children’s Hospital Colorado, Aurora, CO, United States; ^2^ Critical Care, and Pain Medicine, Department of Anesthesiology, Boston Children’s Hospital, Boston, MA, United States; ^3^ COVID-19 Response, Centers for Disease Control and Prevention, Atlanta, GA, United States; ^4^ Departments of Neurology, Boston Children’s Hospital and Harvard Medical School, Boston, MA, United States; ^5^ Division of Pediatric Infectious Diseases, Department of Pediatrics, Vanderbilt University Medical Center, Nashville, TN, United States; ^6^ Division of Pediatric Critical Care, Department of Pediatrics, Cooperman Barnabas Medical Center, Livingston, NJ, United States; ^7^ Division of Pediatric Critical Care Medicine, Medical University of South Carolina, Charleston, SC, United States; ^8^ Department of Pediatrics, University of North Carolina at Chapel Hill Children’s Hospital, Chapel Hill, NC, United States; ^9^ Division of Pediatric Critical Care Medicine, Department of Pediatrics, University of Alabama at Birmingham, Birmingham, AL, United States; ^10^ Section of Critical Care Medicine, Department of Pediatrics, Texas Children’s Hospital, Houston, TX, United States; ^11^ Section of Pediatric Critical Care, Department of Pediatrics, Arkansas Children’s Hospital, Little Rock, AR, United States; ^12^ Division of Pediatric Critical Care Medicine, Department of Pediatrics, Riley Hospital for Children, Indiana University School of Medicine, Indianapolis, IN, United States; ^13^ Division of Critical Care Medicine, Department of Pediatrics, Emory University School of Medicine, Children’s Healthcare of Atlanta, Atlanta, GA, United States; ^14^ Department of Pediatrics, Division of Critical Care, University of California San Francisco, San Francisco, CA, United States; ^15^ Division of Pediatric Critical Care, Department of Pediatrics, Primary Children’s Hospital, University of Utah, Salt Lake City, UT, United States; ^16^ Division of Critical Care Medicine, UCSF Benioff Children’s Hospital, Oakland, CA, United States; ^17^ Division of Pediatric Infectious Disease, Department of Pediatrics, Children’s Mercy Kansas City, Kansas City, MO, United States; ^18^ Department of Anesthesiology and Critical Care, Perelman School of Medicine, Children’s Hospital of Philadelphia, University of Pennsylvania, Philadelphia, PA, United States; ^19^ Department of Pediatrics, Division of Infectious Diseases, Cincinnati Children’s Hospital Medical Center, University of Cincinnati, Cincinnati, OH, United States; ^20^ Division of Infectious Diseases, Department of Pediatrics, University of Mississippi Medical Center, Jackson, MS, United States; ^21^ Division of Critical Care Medicine, Akron Children’s Hospital, Akron, OH, United States; ^22^ Division of Pediatric Critical Care Medicine, Rainbow Babies and Children’s Hospital, Cleveland, OH, United States; ^23^ Division of Pediatric Critical Care Medicine, Department of Pediatrics, C. S. Mott Children’s Hospital and University of Michigan, Ann Arbor, MI, United States; ^24^ Division of Pediatric Critical Care, Department of Pediatrics, Children’s Hospital and Medical Center, Omaha, NE, United States; ^25^ Division of Pediatric Infectious Disease, Department of Pediatrics, AdventHealth for Children, Orlando, FL, United States; ^26^ Division of Pediatric Infectious Diseases, Division of Pediatric Critical Care Medicine, Department of Pediatric and Adolescent Medicine, Mayo Clinic, Rochester, MN, United States; ^27^ Diamond Children’s Banner Children’s Medical Center, University of Arizona, Tucson, AZ, United States; ^28^ Division of Pediatric Critical Care, University of Minnesota Masonic Children’s Hospital, Minneapolis, MN, United States; ^29^ Department of Anesthesiology, Critical Care and Pain Medicine, Boston Children's Hospital, Boston, MA, United States

**Keywords:** post-acute COVID-19 syndrome, COVID-19 post-intensive care syndrome, critical care outcomes, SARS-CoV-2, multisystem inflammatory syndrome in children, MIS-C, COVID-19, pediatrics

## Abstract

**Objective:**

To identify risk factors for persistent impairments after pediatric hospitalization for acute coronavirus disease 2019 (COVID-19) or multisystem inflammatory syndrome in children (MIS-C) during the SARS-CoV-2 pandemic.

**Methods:**

Across 25 U.S. *Overcoming COVID-19* Network hospitals, we conducted a prospective cohort study of patients <21-years-old hospitalized for acute COVID-19 or MIS-C (May 2020 to March 2022) surveyed 2- to 4-months post-admission. Multivariable regression was used to calculate adjusted risk ratios (aRR) and 95% confidence intervals (CI).

**Results:**

Of 232 children with acute COVID-19, 71 (30.6%) had persistent symptoms and 50 (21.6%) had activity impairments at follow-up; for MIS-C (*n* = 241), 56 (23.2%) had persistent symptoms and 58 (24.1%) had activity impairments. In adjusted analyses of patients with acute COVID-19, receipt of mechanical ventilation was associated with persistent symptoms [aRR 1.83 (95% CI: 1.07, 3.13)] whereas obesity [aRR 2.18 (95% CI: 1.05, 4.51)] and greater organ system involvement [aRR 1.35 (95% CI: 1.13, 1.61)] were associated with activity impairment. For patients with MIS-C, having a pre-existing respiratory condition was associated with persistent symptoms [aRR 3.04 (95% CI: 1.70, 5.41)] whereas obesity [aRR 1.86 (95% CI: 1.09, 3.15)] and greater organ system involvement [aRR 1.26 (1.00, 1.58)] were associated with activity impairments.

**Discussion:**

Among patients hospitalized, nearly one in three hospitalized with acute COVID-19 and one in four hospitalized with MIS-C had persistent impairments for ≥2 months post-hospitalization. Persistent impairments were associated with more severe illness and underlying health conditions, identifying populations to target for follow-up.

## Introduction

As of May 17th, 2023, the severe acute respiratory syndrome coronavirus-2 (SARS-CoV-2) pandemic has resulted in over 766 million confirmed cases of coronavirus disease 2019 (COVID-19) globally. In the United States alone, it has led to more than 191,000 pediatric hospitalizations and 2,208 deaths (1–3). Multisystem inflammatory syndrome in children (MIS-C), a post-infectious sequela of SARS-CoV-2 infection emerged early in the pandemic with most patients developing critical illness (4). In the US, there have been over 9,000 hospitalizations for MIS-C, most commonly affecting previously healthy children (4, 5). Fortunately, death due to acute COVID-19 or MIS-C in children is uncommon, with over 98% survival (6–8). The long-term health impacts on these young patients requires additional study.

In a pilot study during the first year of the SARS-CoV-2 pandemic, we reported that more than one in four children and adolescents hospitalized with COVID-19 or MIS-C experienced health impairments beyond two months post-hospital discharge (9). During the time the cohort was admitted, the wild type, Alpha, and Beta SARS-CoV-2 variants were the predominant circulating viral strains (6). Since then, the Delta variant emerged (June–December 2021) followed by Omicron variants (December 2021 to present). These variants resulted in higher numbers of pediatric hospitalizations for acute COVID-19 relative to the earlier SARS-CoV-2 strains (6). However, post-discharge outcomes for children hospitalized with acute COVID-19 or MIS-C during circulation of the Delta and Omicron variants are incompletely described (Supplementary Table S1) (10, 11). This study aims to (a) characterize persistence of symptoms and activity impairment following hospitalization for acute COVID-19 or MIS-C in children and (b) identify risk factors associated with prolonged impairments, including contemporaneous predominant SARS-CoV-2 strain.

## Materials and methods

This multicenter prospective observational cohort study enrolled children and adolescents hospitalized with acute COVID-19 or MIS-C at 25 *Overcoming COVID-19* hospitals (Supplementary Content) (4, 7, 9). Approval was granted by the Boston Children's Hospital's Institutional Review Board (IRB-P00033157), which served as a central institutional review board, and underwent review by the Centers for Disease Control and Prevention (CDC) for compliance with applicable federal law (12). The patient's legal guardian was approached by trained study staff to obtain informed consent and patient assent, when possible, depending on age, developmental capacity, and illness severity.

Children and adolescents (<21-years-old) hospitalized for acute COVID-19 [positive result on a SARS-CoV-2 respiratory test (reverse transcriptase–polymerase chain reaction or antigen) and symptoms related to COVID-19] or MIS-C [per the original 2020 CDC definition (13)] were enrolled between May 12, 2020 and March 17, 2022. We excluded patients with a nosocomial SARS-CoV-2 infection, acquired immune compromise (Supplementary Table S2), end-stage lung disease awaiting transplant, chronic mechanical ventilation support, and limitations of life support because of poor prognosis (9). Subsequent minor modifications made to the exclusion criteria were delineated in Supplementary Table S2. Data were entered into Research Electronic Data Capture (REDCap, Vanderbilt University) hosted at Boston Children's Hospital (14, 15). Race and ethnicity data were collected by participant report and presented per CDC standards (16). Organ system involvement criteria were delineated in Supplementary Table S2. Variable definitions were delineated in Supplementary Table S3.

Primary outcomes were persistent symptoms or activity impairment 2-to-4-months post-hospitalization. Trained study staff conducted telephone interviews and online surveys to collect data regarding persistent symptoms and activity impairments at approximately 1 and 3 months after enrollment. Parents/guardians were asked structured questions to identify current symptoms and newly present activity impairments relative to pre-illness baseline during the 7 days prior to survey completion. Specific interview questions were consistent with prior methods (9) and informed by adult studies (17).

A secondary outcome was change in Pediatric Quality of Life Inventory (PedsQL™) health-related quality of life (HRQL) score which was collected during the second year of the study in patients ≥5 years of age (18). PedsQL™ data were collected by parent-proxy survey at admission (reflecting pre-illness baseline) and parent-proxy survey in children younger than 8-years-old follow-up or child report in patients older than 8-years-old at follow-up. Minimal clinically important difference (MCID) in the PedsQL™ Total Health Summary score, Physical Summary sub-score, and Psychosocial Summary sub-score were a decrease of 4.5 points, 6.92, and 5.49 points, respectively (19). Severe decrease in HRQL was defined as 18 points (4xMCID) below pre-illness Total Health Summary score.

### Statistical analysis

Fisher's exact and Wilcoxon rank sum tests were used to compare differences in categorical and continuous variables, respectively. We performed univariate analyses to identify variables associated with persistent symptoms or impaired activity 2-to-4-months post-hospitalization. Variables considered for inclusion in multivariable models were selected *a priori* based on association with post-COVID complications and clinician expertise (Supplementary Table S3) (9). We assessed for multicollinearity using variance inflation factors and highly multicollinear variables (variance inflation factor ≥2.5) were removed from the final models. Based on univariate and collinearity results of the pre-specified candidate variables, those loosely associated (*P*-value <0.30) with 2- to -4-month outcomes were included in multivariable Poisson regression models using robust variance estimates to determine risk ratios with site as a random intercept. Variables were retained if their removal altered the full model effect estimate by ≥10% or they were significantly associated with the outcome. To determine if risk factors for prolonged impairments differed based on timing of enrollment in children with acute COVID-19, we controlled for periods reflective of primary strain predominance in the multivariable models. We did not include strain-specific period as a variable in the MIS-C models due to decreased incidence of MIS-C during the Delta and Omicron periods. We conducted sensitivity analyses including patients without 2- to 4-month follow-up but without persistent symptoms or activity impairments at 1-month follow-up. We reported risk differences, adjusted risk ratios (aRR), and 95% confidence intervals (CI). *P*-values <0.05 were considered statistically significant. Missing data were not imputed. Analyses were considered exploratory; no adjustments were made for multiple comparisons. Analyses were conducted using R software, version 4.2.2 (Vienna, Austria).

## Results

**Figure 1:**
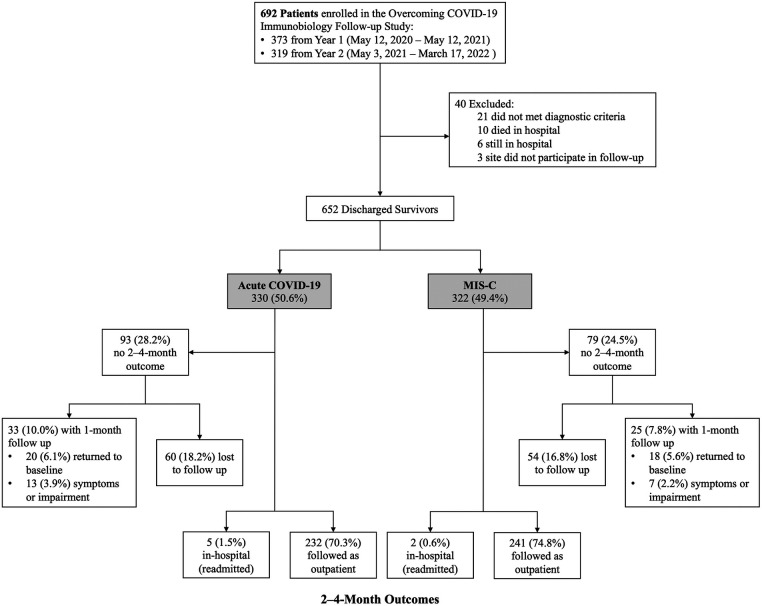
Enrollment and follow-Up of patients hospitalized for acute COVID-19 or MIS-C.

Of the 692 patients enrolled during the study period, 40 were excluded (Figure 1). Of the 652 eligible patients, 330 (50.6%) were hospitalized for acute COVID-19 and 322 (49.4%) MIS-C. Of the acute COVID-19 patients, 232 (70.3%) completed 2- to 4-month surveys median 66 (IQR 62, 72) days after hospitalization, 5 (1.5%) were readmitted at 2- to 4-month follow-up, and 20 (6.1%) had returned to pre-illness baseline at 1-month follow-up. Of the MIS-C patients, 241 (74.8%) completed 2- to 4-month surveys median 65 (IQR 62, 70) days after hospitalization, 2 (0.6%) were readmitted at 2- to 4-month follow-up, and 18 (5.6%) had returned to pre-illness baseline at 1 month follow-up. Patient characteristics did not differ between patients with and without follow-up (Supplementary Table S4). Notably, there was no difference in insurance type or social vulnerability index between patients with and without follow-up. Clinical characteristics among patients admitted with acute COVID-19 differed in that those lost to follow-up had higher illness severity and longer hospitalizations compared with patients who completed follow-up. In contrast, patients admitted with MIS-C who were lost to follow-up had fewer organ systems affected and shorter hospitalizations compared with patients who completed follow-up.

### Patients with acute COVID-19

Of the 232 patients admitted with acute COVID-19 with 2- to 4-month follow-up data, 111 (47.8%) were male, 120 (51.7%) were 13 years or older, and 101 (43.5%) were previously healthy (Table 1). Pre-existing conditions were present in 131 (56.5%) patients including 74 (31.9%) with a pre-existing respiratory condition and 102 (44.0%) with a non-respiratory condition. During hospitalization, 151 (65.1%) were admitted to an intensive care unit (ICU) and 97 (41.8%) were supported with mechanical ventilation. The number of patients enrolled during periods of strain predominance were 127 (54.7%) during Pre-Delta period, 91 (39.2%) during Delta, and 14 (6.0%) during Omicron (Table 1). Among the 18 children with viral co-infections, most were hospitalized during the Omicron-predominant period relative to Delta and Pre-Delta periods (42.9% vs. 8.8% vs. 3.1%, respectively, *p* < 0.001) (Supplementary Table S5). Patients admitted during the Omicron and Delta-predominant periods were more commonly admitted to the ICU (100% and 76.9% vs. 52.8%, *p* < 0.001) and received mechanical ventilation (92.9% and 54.9% vs. 26.8%, *p* < 0.001) compared to patients from the Pre-Delta variant period.

**Table 1:** Acute COVID-19 and MIS-C patient demographic and clinical characteristics.

Characteristic	All COVID-19 patients (*n* = 232)	All MIS-C patients (*n* = 241)
Age group (years), *n* (%)		
<2	50 (21.6)	10 (4.1)
≥2–<5	16 (6.9)	30 (12.4)
≥5–<13	46 (19.8)	123 (51.0)
≥13–<21	120 (51.7)	78 (32.4)
Male sex, *n* (%)	111 (47.8)	145 (60.2)
Race and ethnicity, *n* (%)		
White, non-Hispanic	102 (44)	80 (33.2)
Black, non-Hispanic	47 (20.3)	84 (34.9)
Hispanic or Latino	65 (28.0)	53 (22.0)
Multiple/Other, non-Hispanic	16 (6.9)	17 (7.1)
Unknown	2 (0.9)	7 (2.9)
Social determinants of health, *n* (%)		
Lowest 3rd SVI	45 (19.4)	77 (32.0)
Middle 3rd SVI	73 (31.5)	63 (26.1)
Highest 3rd SVI	114 (49.1)	101 (41.9)
Underlying conditions, *n* (%)		
Previously healthy	101 (43.5)	197 (81.7)
Pre-existing respiratory condition^a^	74 (31.9)	30 (12.4)
Isolated asthma or RAD	40 (17.2)	27 (11.2)
Pre-existing non-respiratory condition^a,b^	102 (44.0)	18 (7.5)
Obesity^c^	96/182 (52.7)	72/231 (31.2)
Received ≥1 SARS-CoV-2 vaccination, *n* (%)	6 (2.6)	11 (4.6)
Vaccine eligible^d^	80 (34.5)	47 (19.5)
Clinical characteristics		
Period of admission, *n* (%)		
Pre-Delta	127 (54.7)	170 (70.5)
Delta	91 (39.2)	49 (20.3)
Omicron	14 (6)	22 (9.1)
Maximum PELOD-2, median (IQR)	1 [0, 3]	3 [2, 4]
Organ systems involved, median (IQR)	3 [2, 4]	5 [4, 6]
ICU admission, *n* (%)	151 (65.1)	198 (82.2)
Mechanical ventilation, *n* (%)^e^	97 (41.8)	65 (27)
Length of MV (days), median (IQR)	0 [0, 1]	0 [0, 3]
Cardiovascular dysfunction, *n* (%)	45 (19.4)	178 (73.9)
Extracorporeal membrane oxygenation, *n* (%)	8 (3.4)	9 (3.7)
ICU LOS (days), median (IQR)	5 [3, 11]	3 [2, 5]
Hospital LOS (days), median (IQR)	6 [3, 12.25]	6 [5, 8]

^a^ Pre-existing condition categories were not mutually exclusive.

^b^ Non-respiratory conditions were: cardiovascular: 40 (17.2%) COVID-19 and 20 (8.3%) MIS-C; neurologic/neuromuscular: 47 (20.3%) COVID-19 and 5 (2.1%) MIS-C; past or current immune compromise that did not meet exclusion criteria (Supplementary Table S2): 9 (3.9%) COVID-19 and 1 (0.4%) MIS-C; gastrointestinal/Hepatic: 43 (18.5%) COVID-19 and 4 (1.7%) MIS-C; hematologic: 16 (6.9%) and 4 (1.7%) MIS-C; renal/urologic: 9 (3.9%) COVID-19 and 2 (8.3%) MIS-C; endocrine: 22 (9.5%) and 6 (2.5%) MIS-C; and metabolic/genetic (not including obesity): 18 (7.8%) COVID-19 and 3 (1.2%) MIS-C.

^c^ Obesity was defined by national reference standards for body mass index if age > 2 years and was considered separately from other pre-existing conditions (20).

^d^ 1 vaccine-eligible COVID-19 patient and 1 vaccine-eligible MIS-C patient had unknown vaccination status.

^e^ Mechanical ventilation duration includes invasive and non-invasive modes of support.

**Figure 2:**
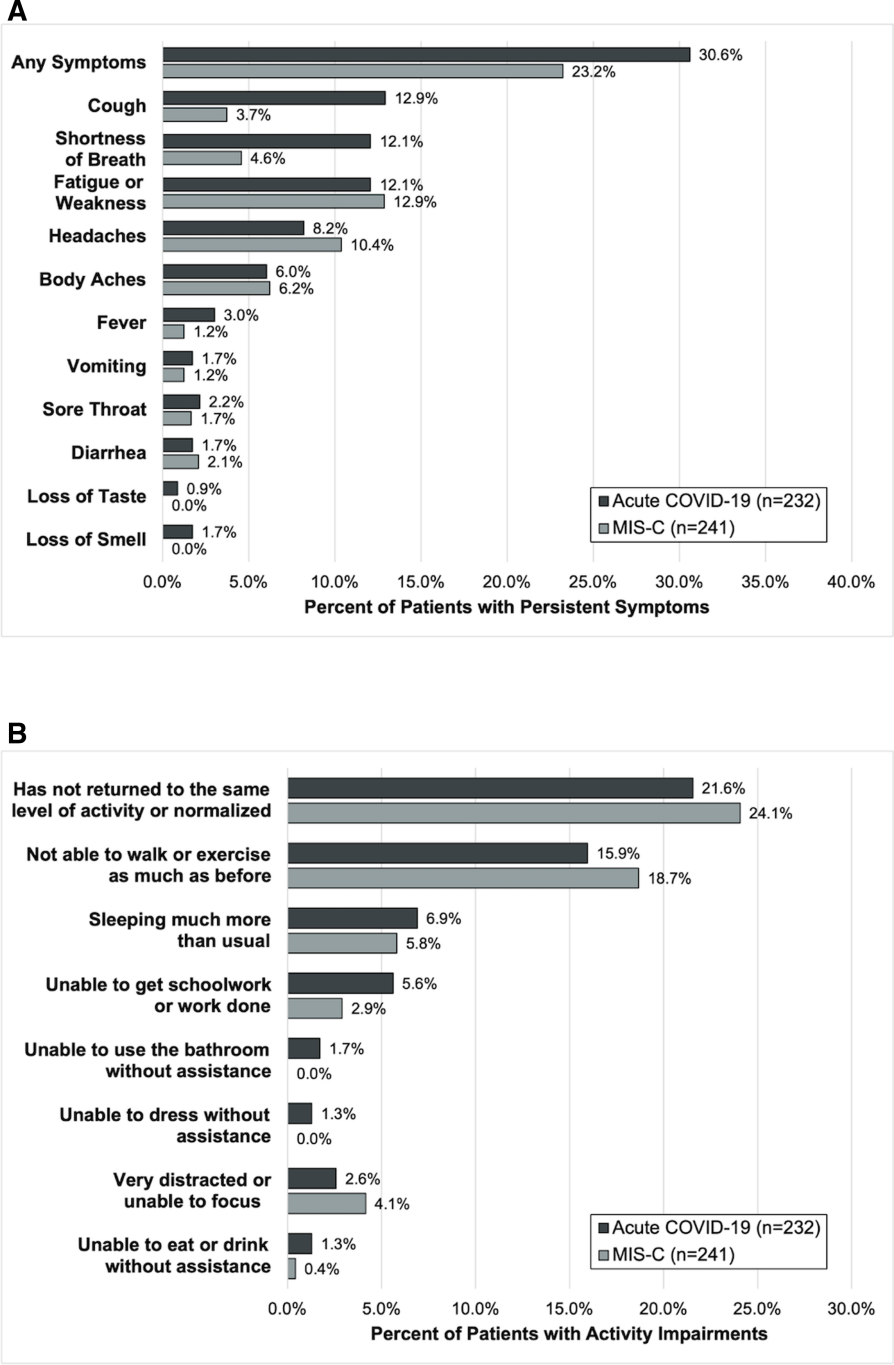
Outcomes of (**A**) persistent symptoms and (**B**) ongoing activity impairment 2- to 4-months after hospitalization among patients admitted for acute COVID-19 and MIS-C.

At 2- to 4-month follow-up, 71 (30.6%) patients had persistent symptoms and 50 (21.6%) had activity impairments (Figure 2 and Supplementary Figure S1). Persistent symptoms were most commonly cough, shortness of breath, and fatigue/weakness. Activity impairments were most often an inability to walk or exercise as much as prior to the illness and sleeping more than usual.

**Figure 3:**
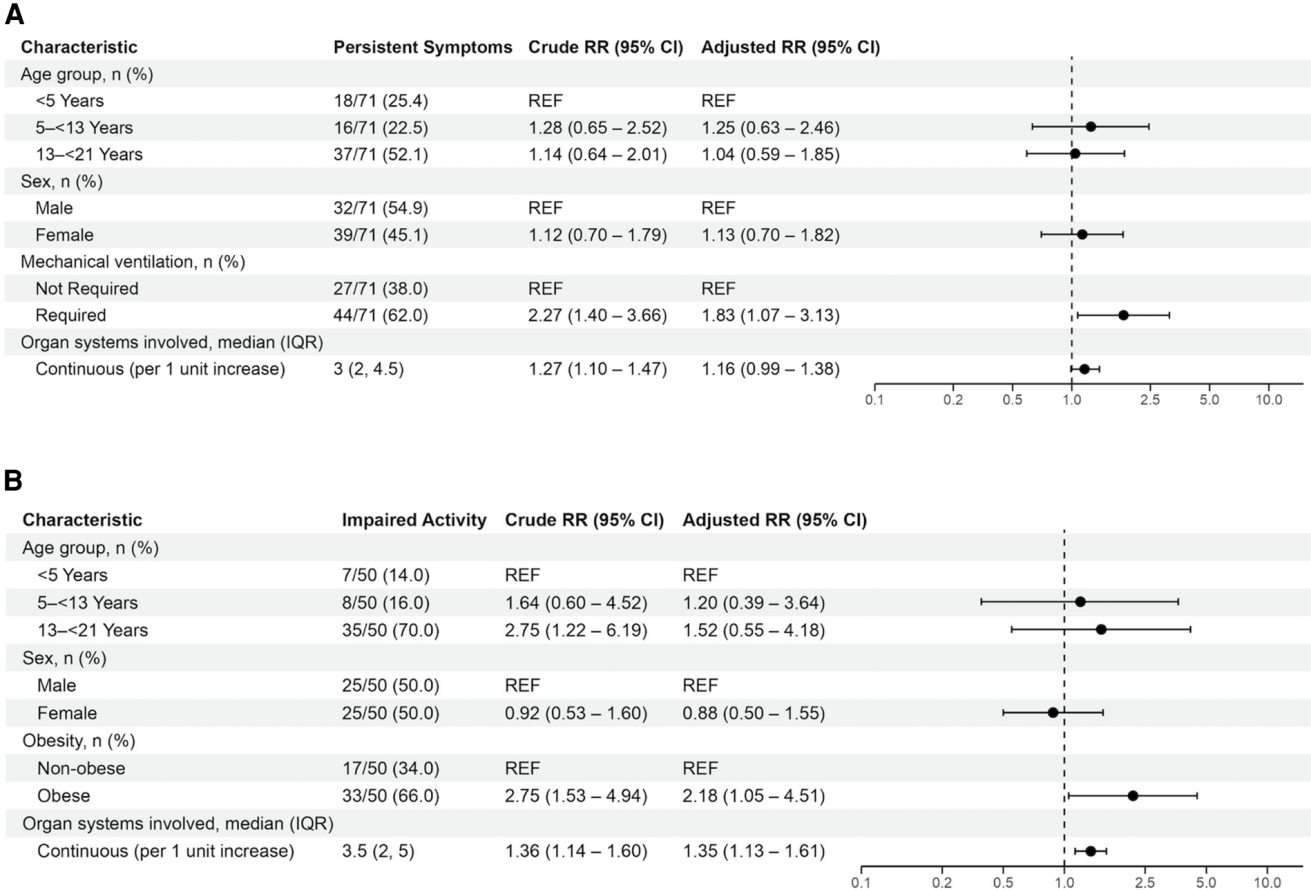
Mixed effects multivariable models evaluating factors associated with (**A**) persistent symptoms or (**B**) activity impairments in patients admitted with acute COVID-19. Variables considered for inclusion in models were age category, sex, SVI category, pre-existing respiratory condition, non-respiratory pre-existing condition, obesity, maximum PELOD-2 score, receipt of mechanical ventilation, duration of mechanical ventilation, cardiovascular dysfunction, and organ systems involved. Patients younger than 2-years-old were considered non-obese.

Patient and clinical characteristics associated with persistent symptoms and activity impairment in univariable analyses were delineated for patients with acute COVID-19 in Supplementary Table S6. In multivariable analyses, receipt of mechanical ventilation was associated with persistent symptoms at 2- to 4-months [aRR 1.83 (95% CI: 1.07, 3.13)] (Figure 3A). Obesity [aRR 2.18 (95% CI: 1.05, 4.51)] and greater organ system involvement [aRR 1.35 (95% CI: 1.13, 1.61)] were associated with persistent activity impairment (Figure 3B). Time period of primary SARS-CoV-2 viral strain was not significant when added to these models (Supplementary Figure S2). Sensitivity analyses including patients without 2- to 4-month follow-up but without persistent symptoms or activity impairments at 1-month follow-up demonstrated similar results (Supplementary Tables S7 and S8).

Health-related quality of life data were available for 70 of 76 (92.1%) patients eligible for HRQL data collection (Supplementary Table S9). At 2- to 4-month follow-up, 25 (35.7%) patients had a clinically important decrease in HRQL score relative to pre-illness baseline including 15 (21.4%) with severely diminished HRQL.

### Patients with MIS-C

Of the 241 patients admitted with MIS-C with 2- to 4-month follow-up data available, 145 (60.2%) were male, 123 (51.0%) were between 5- and 13-years-old, and 197 (81.7%) were previously healthy (Table 1). Forty-four (18.3%) patients had a pre-existing condition; most commonly respiratory (*n* = 30; 68.2%) and this was isolated asthma or reactive airways disease in 27 (90.0%) patients with a respiratory condition. Of the 231 patients 2 years or older, 72 (31.2%) were obese. Most hospitalized children with MIS-C were critically ill: 198 (82.2%) were admitted to an ICU, 65 (27%) were mechanically ventilated, and 178 (73.9%) had cardiovascular dysfunction. The number of patients enrolled during the variant-predominant periods were 170 (70.5%) during Pre-Delta period, 49 (20.3%) during Delta, and 22 (9.1%) during Omicron (Supplementary Table S5).

At follow-up, 56 (23.2%) patients reported persistent symptoms and 58 (24.1%) activity impairments (Figure 2). The most common persistent symptoms were weakness/fatigue and headache and the most reported activity impairment was an inability to walk or exercise the same as before the illness.

**Figure 4:**
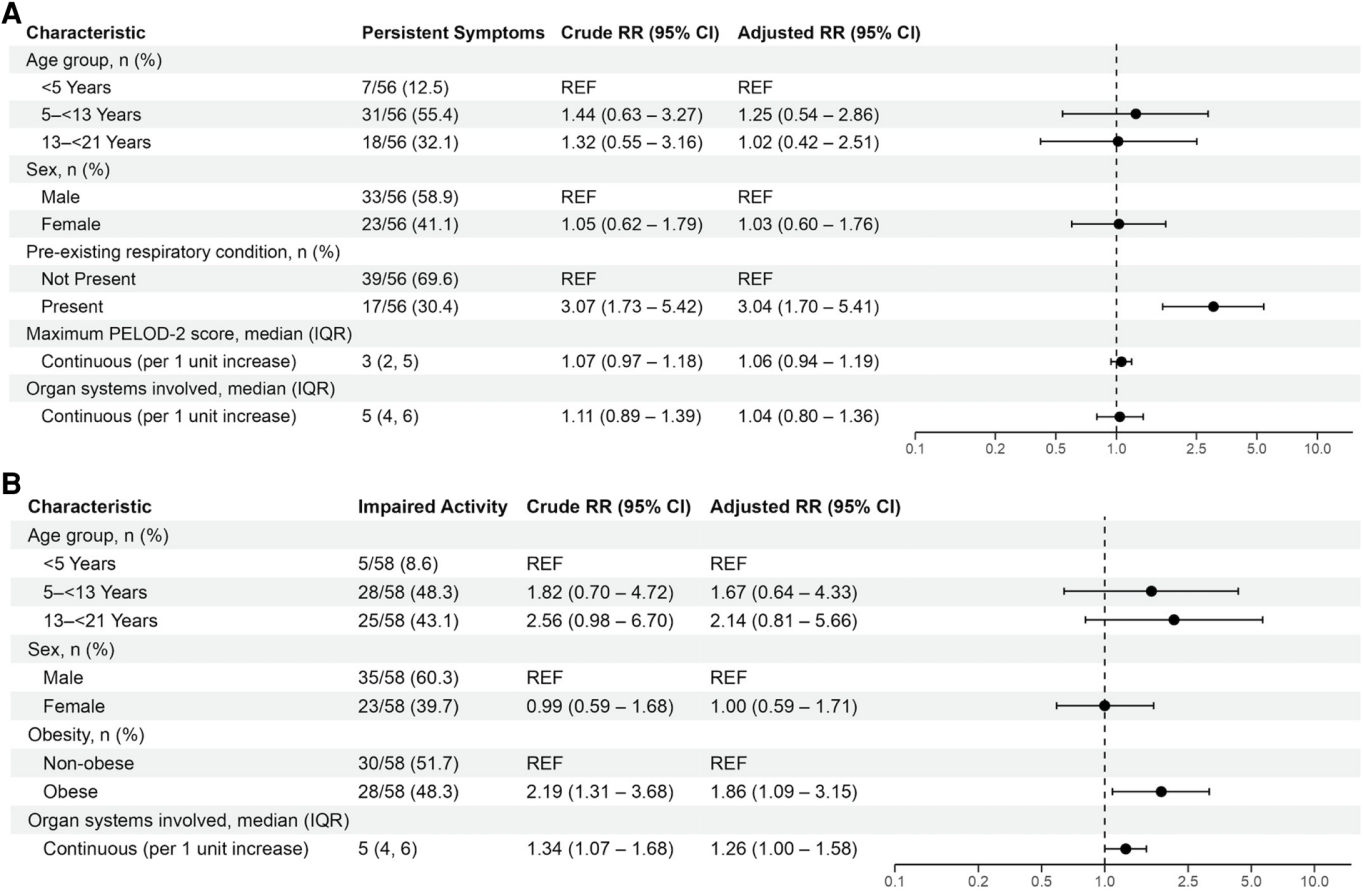
Mixed effects multivariable models evaluating factors associated with persistent symptoms or activity impairments in patients with MIS-C. Variables considered for inclusion in models were age category, sex, SVI category, pre-existing respiratory condition, non-respiratory pre-existing condition, obesity, maximum PELOD-2 score, receipt of mechanical ventilation, duration of mechanical ventilation, cardiovascular dysfunction, and organ systems involved. Patients younger than 2-years-old were considered non-obese.

Patient and clinical characteristics associated with persistent symptoms or impaired activity in univariate analyses were delineated in Supplementary Table S10. In adjusted analyses, having a pre-existing respiratory condition was associated with persistent symptoms [aRR 3.04 (95% CI: 1.70, 5.41)], and obesity [aRR 1.86 (95% CI: 1.09, 3.15)] and number of organ systems affected [aRR 1.26 (1.00, 1.58)] were associated with persistent activity impairment (Figure 4). Sensitivity analyses including patients without 2- to 4-month data but without persistent symptoms or activity impairments demonstrated similar results (Supplementary Tables S11 and S12).

Health-related quality of life data were available for 53 of 57 (93%) patients eligible for HRQL data collection in study year 2 (Supplementary Table S9). Of these 53 patients, 18 (34.0%) patients had a clinically important decrease in HRQL relative to pre-illness baseline including 6 (11.3%) with severely diminished HRQL.

## Discussion

This multicenter study evaluating children admitted with acute COVID-19 or MIS-C demonstrated that nearly one in three children with acute COVID-19 have persistent symptoms, one in five have activity impairments, and one in three have decreased HRQL. Period of admission was not associated with prolonged impairments but obesity and critical illness factors including receipt of mechanical ventilation and number of organ systems affected were associated. Of children presenting with MIS-C, most presented with critical illness. Post-hospitalization morbidity remained high with one in four patients experiencing persistent symptoms or activity impairments and one in three reporting decreased HRQL. In patients admitted with MIS-C, of whom the majority (>80%) were previously healthy, persistent impairments were mostly associated history of asthma or reactive airways disease and/or obesity, as well as the extent of organ system involvement during hospitalization.

Among patients admitted with acute COVID-19, the proportion with persistent symptoms or activity impairments was higher during the periods when Delta and Omicron strains predominated. Notably, patients enrolled during the Delta and Omicron waves experienced more organ system involvement and a greater proportion were mechanically ventilated. After controlling for these factors, admission during these waves was not associated with higher risk of prolonged impairments suggesting that critical illness including supportive strategies and treatments employed during the acute illness were key drivers of long-term outcomes. In a large study conducted using electronic health record data, Rao et al. compared outcomes collected from 59,893 children who tested positive for SARS-CoV-2 with 599,393 who tested negative. Although patients in the study were most commonly tested at an outpatient facility, the study included 674 SARS-CoV-2 positive and 13,847 SARS-CoV-2 negative patients who were admitted to an ICU. In adjusted analyses, they identified that ICU admission, regardless of SARS-CoV-2 positivity was associated with a 1.35 times higher hazard of post-acute sequelae and this risk increased to 2.11 times when a patient was SARS-CoV-2 positive (21). These findings, in conjunction with our study and others that have identified critical illness as a risk factor for prolonged impairments, suggest that persistence of ICU-related impairments is likely to be important outcomes for children admitted with acute COVID-19 (22–26).

Interestingly, the distribution of symptoms and complications and prevalence of co-infections also changed during our study period. Reports of other U.S. pediatric cohorts suggested that, as the pandemic progressed, younger children were increasingly admitted with acute COVID-19 (6). Our study was characterized by a similar pattern. Notably, the Omicron variant replicates more robustly in the conducting airways than lung parenchyma placing younger children at higher risk of illnesses such as severe croup due to their smaller airway diameter (27–29). Additionally, there were very few other viruses co-detected during the initial months of the pandemic whereas co-detection increased during the Delta and Omicron waves. This was noted in our population in whom viral co-detection occurred in 42.9% of children with acute COVID-19 during the Omicron wave, corresponding with resurgence of other acute respiratory viral illnesses after easing of COVID-19 restrictions. Importantly, co-infections have been reported to be associated with more severe outcomes, particularly in younger children (6). These changes which are inherent to viral strain as well as the epidemiology of circulating infections may place children at higher risk of severe disease and prolonged impairments associated with acute COVID-19 over time.

To date, the few studies evaluating post-discharge outcomes in children with MIS-C aside from cardiac outcomes have reflected outcomes of patients admitted during the Pre-Delta period (9, 30–35). A small cohort study conducted in India evaluated persistent symptoms a median of 5 months after MIS-C in 34 children, the majority of whom were admitted to an ICU (30). In this study, one in ten experienced prolonged symptomatology. In a retrospective study of 45 children with severe MIS-C and clinical follow-up 3 months after discharge, 18% experienced abdominal pain, 18% cardiac involvement, 16% pulmonary symptoms, and 13% neuropsychiatric findings (32). Penner et al. reported a similar proportion of approximately one in five patients with impaired physical and emotional functioning among 46 children with MIS-C when evaluated 6 months after illness (31). Kahn et al. reported that one in three patients had abnormal findings based on patient history, most commonly reporting fatigue 3 months post-illness (33). When restricted to the 15 critically ill patients in this cohort, the proportion with an abnormal history increased to 50%. In a cohort of 49 children admitted with MIS-C in the Netherlands evaluated 4 months after discharge, nearly half reported impaired exercise tolerance and more than half demonstrated worse function in visual memory and attention, more emotional and behavioral problems, and lower quality of life scores relative to population norms (35). Our study identified that patient and hospitalization factors such as pre-existing respiratory conditions, obesity, and organ system involvement were consistently associated with prolonged impairments. Taken together, these studies suggest that in patients with MIS-C, the majority of whom are previously healthy, at least 20%–30% are likely to experience prolonged impairments during the months after illness.

The post-acute sequelae described in our cohort offer valuable insights into the post-COVID conditions experienced by children and young adults. Impairments delineated in the acute COVID-19 cohort represent “long COVID” which is defined as ongoing, relapsing, or new symptoms or conditions present 30 or more days after infection (36). While pediatric studies delineating the recovery trajectory of children with multisystem involvement are lacking, adult studies suggest improvement during the post discharge year but that symptoms may persist in some patients beyond one year post-illness (37). Regarding children hospitalized with MIS-C, the impairments described for this cohort represent sequelae of a post-infectious complication of COVID, which is not considered “long COVID” but remains an important outcome for children infected with SARS-CoV-2. While cardiac abnormalities are likely to resolve during the post-acute recovery period, the trajectory of the multisystem impairments requires further investigation (38).

This study has important limitations. First, the patients with follow-up data differed from those lost to follow-up based on important characteristics including illness severity. Illness severity was higher among patients admitted with acute COVID-19 who were lost to follow-up whereas the opposite was true among patients admitted with MIS-C. Second, this study predominantly included patients with severe or critical illness and did not include a matched control group of less severely ill or SARS-CoV-2 negative patients. Third, strain-predominant periods represent the epidemiology of circulating SARS-CoV-2 viral strains and not patient-level variant data or virus-specific co-infection data. Fourth, we were unable to control for vaccination status given the low proportion of vaccinated patients. Fifth, treatments provided to patients were likely to differ across institutions and time periods and our study did not have the power to assess the impact of treatments or interventions aimed at preventing morbidities but this has been explored in a separate manuscript focused on MIS-C treatment (34). Sixth, in patients 8 years or older, HRQL data was collected from the guardian (pre-illness baseline) and subsequently from the patient (post-discharge) which may have affected comparisons. Lastly, the data element related to insurance type was limited as we were unable to determine whether medicaid was administered through a government or commercial plan.

In this cohort of predominantly critically ill children admitted for SARS-CoV-2-related complications, mortality was very low but many patients suffered from morbidities during the months after hospitalization. One in three patients admitted with acute COVID-19 had prolonged impairments particularly those with multiple organ system involvement and need for mechanical ventilation as well as those with obesity. Although >80% of children with MIS-C were previously healthy, one in four children admitted with MIS-C also experienced prolonged impairments, again related to multiple organ system involvement and obesity as well as pre-existing respiratory conditions. Further studies are needed to delineate the impact of vaccination on prolonged impairments in children hospitalized with critical illness due to SARS-CoV-2 and the persistence and recoverability of these impairments.

## Data Availability

The datasets presented in this article are not readily available because Participants did not consent to sharing of their data beyond this study. Requests to access the datasets should be directed to adrienne.randolph@childrens.harvard.edu.
