# Supplementary material for: Risk factors for health impairments in children after hospitalization for acute COVID-19 or MIS-C
Source: Front Pediatr. 2023 Oct 18;11:1260372. doi: 10.3389/fped.2023.1260372 (PMC10619659; doi:10.3389/fped.2023.1260372)
Supplement: Supplementary file 1 [file Table1.docx]

**Supplemental Digital Content** for Risk Factors for Health Impairments in Children after Hospitalization for Acute COVID-19 or MIS-C

**Table of Contents:**

| **Table S1** – Previously Published Reports of Post-Discharge Outcomes of Children with Critical Illness due to Acute COVID-19 or MIS-C | Pages 2–3 |
| --- | --- |
| **Overcoming COVID-19 Investigators** | Pages 4-5 |
| **CDC Response Team on Overcoming COVID-19** | Page 5 |
| **Table S2 –** Supplemental Methods | Pages 6-7 |
| **Table S3** – Variables Considered for Inclusion in Multivariable Models | Page 8 |
| **Table S4** – Comparison of Acute COVID-19 and MIS-C Patient and Clinical Characteristics Among Patients With and Without Post-Discharge Follow Up | Pages 9-10 |
| **Table S5** – Comparison of Acute COVID-19 and MIS-C Patient and Clinical Characteristics by Strain-Predominant Time Period | Pages 11-13 |
| **Figure S1** – Outcomes of (A) Persistent Symptoms and (B) Ongoing Activity Impairment 2 to 4 Months After Hospitalization Among Patients Admitted for Acute COVID-19 | Page 14 |
| **Table S6** – Patient and Clinical Factors Associated With Persistent Symptoms or Activity Impairment at 2 to 4 Months After Admission in Patients with Acute COVID-19 | Pages 15-16 |
| **Figure S2** – Mixed Effects Multivariable Models Evaluating Factors Associated with Persistent Symptoms or Activity Impairments in Patients with Acute COVID-19 Including Variable for Strain-Predominant Time Period | Pages 17-18 |
| **Table S7** – Patient and Clinical Factors Associated With Persistent Symptoms or Activity Impairment at 2 to 4 Months After Admission in Patients with Acute COVID-19 Including Patients Missing 2-4-Month Follow-up But Normalized by 1 Month (sensitivity analysis) | Pages 19-20 |
| **Table S8 –** Mixed Effects Multivariable Models Evaluating Factors Associated with Persistent Symptoms or Activity Impairments in Patients with Acute COVID-19 Including Variable for Strain-Predominant Time Period and Patients without 2-4-Month Follow-Up Data but Normalized at 1 Month (sensitivity analysis) | Pages 21-22 |
| **Table S9** – Health-Related Quality of Life Prior to Illness and at Follow-up Among Patients with Acute COVID-19 and MIS-C Enrolled during the Second Year of the Study | Page 23 |
| **Table S10** – Patient and Clinical Factors Associated With Persistent Symptoms or Activity Impairment at 2 to 4 Months After Admission in Patients with MIS-C | Page 24-25 |
| **Table S11** – Patient and Clinical Factors Associated With Persistent Symptoms or Activity Impairment at 2 to 4 Months After Admission in Patients with MIS-C Including Patients Missing 2-4-Month Follow-up But Normalized by 1 Month (sensitivity analysis) | Page 26 |
| **Table S12 –** Mixed Effects Multivariable Models Evaluating Factors Associated with Persistent Symptoms or Activity Impairments in Patients with MIS-C Including Patients without 2-4-Month Follow-Up Data but Normalized at 1 Month (sensitivity analysis) | Page 27 |
| **References** | Page 28 |

**Table S1** – Previously Published Reports of Post-Discharge Outcomes of Children with Critical Illness due to Acute COVID-19 or MIS-C

| **Acute COVID-19** | **Cohort (% hospitalized), Enrollment period** | **Evaluation** | **Findings** |
| --- | --- | --- | --- |
| Asadi-Pooya (1)  Iran | N=58 (100%). 10/58 patients in the ICU  Feb 2020 – Nov 2020 | phone follow-up at 3 months post dc, symptoms, and exercise intolerance | 45% of patients had long COVID, 21% fatigue, 12% short of breath, 12% exercise intolerance, weakness, and walking intolerance in about 10%. Older age, muscle pain on admission, ICU admission associated with prolonged symptoms. |
| Osmanov(2)  Russia | N=518 (100%), <3% of population (n=14) had critical illness  April 2020 – Aug 2020 | phone follow-up median 256 d post dc, Symptoms (ISARIC) | 24% had persistent symptoms: fatigue (10.7%), sleep disturbance (6.9%), sensory problems (5.6%). Risk factors: age > 6yo and history of allergic disease |
| Rao(3)  U.S. | 59,893 COVID+, 1,656 (2.8%) tested + during a hospitalization.  March 2020 – Oct 2021 | Electronic health record | Hazard of post-acute sequelae of SARS-CoV-2 infection increased with: ICU with positive SARS-CoV-2 test (aHR 2.11 [1.91, 2.34]) and ICU with negative SARS-CoV-2 test (aHR: 1.35 [1.32, 1.39]). |
| Baptista de Lima(4)  Portugal | 237 children with SARS-CoV-2 positive test during an ED or visit or hospitalization, 3% of cohort critically ill.  March 2020 – Sep 2021 | Electronic health record evaluation of data collected during follow-up appointments | Adolescents and patients with critical illness on admission were more likely to report persistent symptoms at 24 weeks. |
| Pazukhina(5)  Russia | n=360 (100%) hospitalized children including 12 admitted to an ICU  Apr 2020- Aug 2020 | Phone interviews 6- and 12-months post-discharge | Post-covid conditions at 6 months post-discharge: 9% with fatigue, 5% dermatologic, 4% neurocognitive, 4% sleep related. At 12 months, this decreased to 4%, 2%, 2% and 1%. Risk factors for post-COVID conditions were pre-existing neurologic comorbidities and history of allergic respiratory diseases. |
| Valenzueala(6)  Chile | n=216 (100%)  March 2020 – Dec 2021 | Data recorded in outpatient follow-up visits within 6 months of discharge. | Obesity was associated with ICU admission, higher need for respiratory support as well as post-acute sequelae (> 4 weeks post-infection) including dyspnea and muscle weakness. |
| **MIS-C** |  |  |  |
| Penner (7)  Single center, UK | N=46 (100%)  April 2020 – Sep 2020 | follow-up at 6 weeks and 6 months, functional outcome and labs | 6 months: inflammation had resolved, echocardiograms nearly all normal. Minimal functional impairment. 45% of patients with very poor 6-min walk test, 20% of pts with severe emotional difficulties. |
| Kahn (8)  Sweden | N=133/177 eligible in a national population based registry, 89 (67%) with 8 week follow-up data  17% (n=15) patients in the 8-week follow-up cohort treated in ICU and 83% (n=74) treated in ward or outpatient setting  Dec 2020 – May 2021 | Symptoms and echo, f-up at 2 weeks and 8 weeks | 36% of patients had persistent symptoms 8 weeks after MIS-C, 5% had abnormal echocardiograms. Symptoms most frequently fatigue (14%) GI and ha (8%), muscle or joint weakness or pain (7%) or decreased exercise capacity (6%). Older children and those in the ICU were more likely to report symptoms and have abnormal cardiac results. |
| Awasthi (9)  India | 40 children with MIS-C, 85% admitted to the ICU, 34 with follow-up data.  Sep 2020 – Jan 2021 | Follow-up evaluation. | 11.8% reported symptoms at follow-up which occurred a median of 5 months after hospitalization. |
| Sezer (10)  Turkey | 123 patients admitted for MIS-C.  July 2020 - July 2021 | Retrospective review of electronic health record | Follow-up occurred a median 7.8 months after hospitalization. 6.2% were readmitted to the hospital. The most common reasons for admission to the hospital were recurrent abdominal pain (14.2%), cardiac findings (14.2%), pulmonary symptoms (8%), fever (7.1%), neuropsychiatric findings (6.2%) and hypertension (3.5%) |
| Son (11)  U.S. | 186 patients admitted with MIS-C.  May 2020 – May 2021 | Prospective cohort study | Specifically evaluated steroid treatment after discharge. Identified frequent weight gain after discharge.  Readmission occurred in 7 (3.8%) patients. No difference in resolution of clinical symptoms based on whether post-discharge steroid duration < or > 3 weeks. |
| Otten (12)  Netherlands | 49 children with MIS-C  March 2020 – June 2021 | Prospective cohort study | Children evaluated median 4 months after discharge.  43% reported impaired exercise tolerance and 20% worse sleep behavior.  Found to have normal intelligence and verbal memory scores (compared with population norms).  29 (59%) had extensive testing that demonstrated worse function in visual memory, sustained attention, and planning. They also had more emotional and behavioral problems, lower quality of life scores (physical and school functioning domains), and increased fatigue compared with population norms. |

**Overcoming COVID-19 Study Group Investigators**

(listed in PubMed, and ordered by U.S. State)

The following study group members were all closely involved with the design, implementation, and oversight of the Overcoming COVID-19 study and their enrolling hospital site is noted.

**Alabama:** Children’s of Alabama, Birmingham. Michele Kong, MD; Meghan Murdock, RN, Heather Kelley, RN; Candice Colston

**Arizona:** University of Arizona, Tucson. Mary Glas Gaspers, MD; Katri V. Typpo, MD.

**Arkansas:** Arkansas Children’s Hospital, Little Rock. Ronald C. Sanders Jr., MD, MS; Katherine Irby, MD.

**California:** UCSF Benioff Children’s Hospital Oakland, Oakland. Natalie Z. Cvijanovich, MD.

**California:** UCSF Benioff Children’s Hospital, San Francisco. Matt S. Zinter, MD.

**Colorado:** Children’s Hospital Colorado, Aurora. Aline B. Maddux, MD, MSCS., Emily Port, BA, PMP; Rachel Mansour, BSN, RN, CPN; Sara Shankman, DNP, CPNC-AC.

**Florida:** Holtz Children’s Hospital, Miami. Brandon M. Chatani, MD.

**Georgia:** Emory University, Children's Healthcare of Atlanta at Egleston, Atlanta. Keiko M. Tarquinio, MD; Kaitlin Jones, RN.

**Indiana:** Riley Hospital for Children, Indianapolis. Courtney M. Rowan, MD, MS.

**Massachusetts:** Boston Children’s Hospital, Boston. Adrienne G. Randolph, MD; Margaret M. Newhams, MPH; Cameron C. Young, BS; Suden Kucukak, MD; Caitlin Rollins, MD; Tanya Novak, PhD; Janet Chou, MD; Mary Beth Son, MD; Julia Clarke, BA; Brooke Sens, BSN, RN; Eve Listerud; Sabrina Chen, BS; Kasey Stewart.

**Michigan:** University of Michigan CS Mott Children’s Hospital, Ann Arbor. Heidi R. Flori, MD, FAAP; Mary K. Dahmer, PhD.

**Minnesota:** University of Minnesota Masonic Children’s Hospital, Minneapolis, Janet R. Hume, MD, PhD.

**Minnesota:** Mayo Clinic, Rochester. Emily R. Levy, MD; Supriya Behl, MSc; Noelle M. Drapeau, BA.

**Mississippi:** Children’s Hospital of Mississippi, Jackson. Charlotte V. Hobbs, MD; Lora Martin, MSN, FNP; Lacy Malloch, BS; Maygan Martin, RN; Kayla Patterson, MS; Cameron Sanders, BS; Kengo Inagaki, MD; Sarah McGraw, DNP; Anita Dhanrajani, MD

**Missouri:** Children’s Mercy Kansas City, Kansas City. Jennifer E. Schuster, MD, Abigail Kietzman BS, ACRP-CP; Shannon Hill RN, BSN.

**Nebraska:** Children’s Hospital & Medical Center, Omaha. Melissa L. Cullimore, MD, PhD; Russell J. McCulloh, MD.

**New Jersey:** Cooperman Barnabas Medical Center, Livingston. Shira J. Gertz, MD.

**North Carolina:** University of North Carolina at Chapel Hill, Chapel Hill. Stephanie P. Schwartz, MD; Tracie C. Walker, MD.

**Ohio:** Akron Children’s Hospital, Akron. Ryan A. Nofziger, MD.

**Ohio:** Cincinnati Children’s Hospital, Cincinnati. Mary Allen Staat, MD, MPH.

**Ohio:** Rainbow Babies and Children’s Hospital, Cleveland. Steven Shein, MD; Rajashri Rasal, MPH.

**Pennsylvania:** Children’s Hospital of Philadelphia, Philadelphia. Julie C. Fitzgerald, MD, PhD, MSCE; Ryan Burnett; Jenny Bush.

**South Carolina:** MUSC Children’s Health, Charleston. Elizabeth H. Mack, MD, MS.

**Tennessee:** Monroe Carell Jr. Children’s Hospital at Vanderbilt, Nashville. Natasha B. Halasa, MD, MPH. Meena Golcha, MD, Laura Stewart, PhD.

**Texas:** Texas Children’s Hospital, Houston. Laura L. Loftis, MD.

**Utah:** Primary Children’s Hospital, Salt Lake City. Hillary Crandall, MD PhD; Krow Ampofo, MBChB.

**CDC COVID-19 Response Team on Overcoming COVID-19:** Manish M. Patel, MD, MPH; Leora R. Feldstein, PhD, MSc; Mark W. Tenforde, MD PhD; Ashley M. Jackson MPH, Angela Campbell, MD, MPH; Laura D. Zambrano, PhD.

**Table S2 –** Supplemental Methods

| **Acquired Immune Compromise** includes HIV; active chemotherapy; active cancer; immune modulating therapy for rheumatologic, immune, or oncologic disorders or after transplantation to prevent rejection. |
| --- |
| **Additional exclusions after March 31, 2021:**   - Patients with chronic underlying cardiac condition with ejection fraction at <55% at baseline.   **For acute COVID-19 patients:**   - Previously diagnosed inflammatory bowel disease or other chronic inflammatory condition of the gastrointestinal tract. - Chronic respiratory disorders including interstitial lung disease, cystic fibrosis, need for chronic oxygen or mechanical ventilator support, or prior hospital admission for asthma in the past year. - Patient is a ward of the court or in Department of Social Services (DSS) custody |
| **Organ System Involvement Criteria**  *One criterion must be met to constitute organ system involvement.*  **Cardiovascular**   - Shock requiring vasopressors. - Cardiac arrest - Pulmonary edema due to left heart failure - Receipt of vasoactive infusions (at any time during hospitalization)   - Dopamine, Dobutamine, Epinephrine, Norepinephrine, Phenylephrine, Milrinone, Vasopressin (for hypotension, not diabetes insipidus) - Myocarditis or cardiac dysfunction diagnosed during hospital stay - Coronary artery aneurysm   - Z Score of LAD or RCA ≥ 2.5 - Left ventricle ejection fraction (LVEF) < 55% - Arrhythmia - Elevated troponin (based on site-specific cutoff) - Aortic valve regurgitation noted on ECHO - Mitral valve regurgitation noted on ECHO - Pericarditis or pericardial effusion - Cardiopulmonary resuscitation - Maximum BNP or NT-proBNP > 400 pg/mL |
| **Respiratory**   - Respiratory failure requiring support - Receipt of any oxygen support at any time during hospitalization - Pulmonary infiltrates on chest radiograph - Pneumothorax - Pleural effusion - Chest-tube or drainage required - Pulmonary hemorrhage - Lower respiratory infection - Severe bronchospasm requiring continuous bronchodilators |
| **Renal**   - Dialysis or hemofiltration during hospitalization - Elevated creatine (acute kidney injury)   - Less than 4 weeks: >1.5 mg/dL   - 4 weeks to <1 year: >0.6 mg/dL   - 1 year to 10 years: >1.05 mg/dL   - 11 years and older: >1.5 mg/dL |

| **Neurological**   - Suspected central nervous system infection - Stroke or intracranial hemorrhage (at presentation or during hospitalization) - Seizure (at presentation or during hospitalization) - Coma or unresponsive - Receipt of neurodiagnostic imaging or evaluation (CT, MRI, or LP) - Encephalitis - Decreased hearing - Decreased vision - Iritis or uveitis |
| --- |
| **Gastrointestinal**   - Appendicitis - Diarrhea (at presentation or during hospitalization) - Abdominal pain (at presentation or during hospitalization) - Gallbladder hydrops or edema - Pancreatitis - Hepatitis - Nausea/Loss of appetite at presentation - Vomiting at presentation |
| **Hematological**   - Anemia   - Less than 4 weeks: hemoglobin <10 g/dL   - Greater than 4 weeks old: hemoglobin <9 g/dL - Minimum white blood cells <4 x 10^3^ cells/µL - Minimum platelets <150 x 10^3^ cells/µL - Deep vein thrombosis - Pulmonary embolism - Hemolysis - Bleeding - Ischemia of an extremity |

| **Dermatological/Mucocutaneous**   - Bilateral conjunctival injection - Oral mucosal changes (erythema of lips or oropharynx, strawberry tongue, or drying or fissuring of the lips) - Peripheral extremity changes (edema, erythema, or generalized or periungual desquamation) - Skin rash/Skin ulcers - 'COVID toes' (changes or purple spots on toes) - Swollen red cracked lips - Erythema of palms or soles - Edema of hands or feet - Periungual (nails) desquamation - Conjunctivitis - Peripheral gangrene |
| --- |

**Table S3** – Variables Considered for Inclusion in Multivariable Models

| **Variable, name (levels)** | | |
| --- | --- | --- |
| Age Category (<2 Years, 2–<5 Years, 5–<13 Years, 13–<21 Years)^a^ | | |
| Sex^a^ | | |
| Social Vulnerability Index Category (Lowest 3^rd^, Middle 3^rd^, Highest 3^rd^)^b^ | | |
| Pre-Existing Respiratory Condition | | |
| Non-Respiratory Pre-Existing Condition | | |
| Obesity^c^ | | |
| Maximum PELOD-2 Score (continuous)^d^ | | |
| Organ systems involved (continuous) | | |
| Mechanical Ventilation (Invasive or non-invasive)^e^ | | |
| Days of Mechanical Ventilation (continuous) | | |
| Cardiovascular Dysfunction^f^ | | |
| Study Period based on Strain Predominance | | |
| Study Period | Acute COVID-19 | MIS-C^g^ |
| Pre-Delta | Prior to 6/26/2021 | Prior to July 10^th^, 2021 |
| Delta | 6/26/2021- 12/17/2021 | 7/10/2021 – 12/31/2021 |
| Omicron | After 12/18/2021 | After 1/1/2021 |

^a^Age and sex were included in all multivariable models.

^b^Social vulnerability index (SVI) was determined using the first four digits of a patient’s home zip code during 2020 and the full address during 2021 and 2022 (13).

^c^Obesity was defined by national reference standards for body mass index if aged ≥2 years and those < 2 years were considered non-obese (14).

^d^Pediatric Logistic Organ Dysfunction 2 (PELOD-2) scores were collected on hospitalization days 1-7, 10, 14, 18, 22, 28, 35 and 42 to identify maximum PELOD-2 score (15).

^e^Mechanical ventilation was defined as receipt of invasive or non-invasive mechanical ventilation.

^f^Cardiovascular dysfunction was defined as measurement of left ventricular ejection fraction ≤55% on at least one echocardiogram or vasopressor-dependent shock.

^g^For MIS-C classifications, time periods started approximately two weeks after acute COVID-19 time periods. Due to the small numbers of patients with MIS-C during the Delta and Omicron time periods, this value was not included in the MIS-C multivariable models.

**Table S4** – Comparison of Acute COVID-19 and MIS-C Patient and Clinical Characteristics Among Patients With and Without Post-Discharge Follow Up

| **Characteristic** | **COVID-19 with Follow Up^a^** | **COVID-19 without Follow Up** | **P-value** | **MIS-C with Follow Up^a^** | **MIS-C without Follow Up** | **P-value** |
| --- | --- | --- | --- | --- | --- | --- |
| n (%) | 257/330 (77.9) | 73/330 (22.1) |  | 261/322 (81.1) | 61/322 (18.9) | NA |
| **Age Group (years), n (%)** | | | | | | |
| <2 | 56 (21.8) | 12 (16.4) | 0.67 | 9 (3.4) | 2 (3.3) | 0.20 |
| 2-<5 | 18 (7) | 4 (5.5) |  | 34 (13.0) | 7 (11.5) |  |
| 5-<13 | 51 (19.8) | 18 (24.7) |  | 134 (51.3) | 40 (65.6) |  |
| 13-<21 | 132 (51.4) | 39 (53.4) |  | 84 (32.2) | 12 (19.7) |  |
| Male sex, n (%) | 120 (46.7) | 40 (54.8) | 0.23 | 155 (59.4) | 36 (59.0) | 1.00 |
| **Race and Ethnicity, n (%)** | | | | | | |
| White, non-Hispanic | 110.253 (42.9) | 28 (38.4) | 0.66 | 86 (33) | 19 (31.1) | 0.95 |
| Black, non-Hispanic | 54.741 (21.3) | 17 (23.3) |  | 92 (35.2) | 24 (39.3) |  |
| Hispanic or Latino | 72.217 (28.1) | 21 (28.8) |  | 58 (22.2) | 13 (21.3) |  |
| Multiple/Other, non-Hispanic | 17.733 (6.9) | 5 (6.8) |  | 18 (6.9) | 3 (4.9) |  |
| Unknown | 2.056 (0.8) | 2 (1.2) |  | 7 (2.7) | 2 (3.3) |  |
| **Social Determinants of Health** | | | | | | |
| Public Insurance, n (%) | 162 (63) | 48 (65.8) | 0.18 | 140 (53.6) | 32 (52.5) | 0.26 |
| Private Insurance, n (%) | 90 (35) | 21 (28.8) |  | 110 (42.1) | 29 (47.5) |  |
| Unknown Insurance, n (%) | 5 (1.9) | 4 (5.5) |  | 11 (4.2) | 0 (0) |  |
| Social Vulnerability Index (SVI), median (IQR) | 0.65 [0.386, 0.839] | 0.67 [0.46, 0.76] | 0.58 | 0.56 [0.25, 0.78] | 0.47 [0.24, 0.84] | 0.92 |
| Lowest 3^rd^ SVI, n (%) | 50 (19.5) | 15 (20.5) | 0.97 | 84 (32.2) | 19 (31.1) | 0.84 |
| Middle 3^rd^ SVI, n (%) | 81 (31.5) | 22 (30.1) |  | 68 (26.1) | 18 (29.5) |  |
| Highest 3^rd^ SVI, n (%) | 126 (49) | 36 (49.3) |  | 109 (41.8) | 24 (39.3) |  |
| **Underlying Conditions, n (%)** | | | | | | |
| Previously Healthy | 109 (42.4) | 29 (39.7) | 0.79 | 213 (81.6) | 49 (80.3) | 0.86 |
| Pre-existing Respiratory Condition^b^ | 84 (32.7) | 21 (28.8) | 0.57 | 35 (13.4) | 8 (13.1) | 1.00 |
| Isolated asthma or reactive airways disease | 44 (17.1) | 17 (23.3) | 0.24 | 31 (11.9) | 8 (13.1) | 0.83 |
| Pre-existing non-respiratory condition^b^ | 119 (46.3) | 34 (46.6) | 1.00 | 20 (7.7) | 1 (1.6) | 0.14 |
| Cardiovascular | 26 (10.1) | 5 (6.8) | 0.50 | 4 (1.5) | 0 (0) | 1.00 |
| Neurologic/Neuromuscular | 51 (19.8) | 15 (20.5) | 0.87 | 6 (2.3) | 1 (1.6) | 1.00 |
| Immunocompromised | 11 (4.3) | 0 (0) | 0.13 | 2 (0.8) | 0 (0) | 1.00 |
| Gastrointestinal/Hepatic | 46 (17.9) | 12 (16.4) | 0.86 | 5 (1.9) | 0 (0) | 0.59 |
| Hematologic | 19 (7.4) | 5 (6.8) | 1.00 | 4 (1.5) | 1 (1.6) | 1.00 |
| Renal/Urologic | 9 (3.5) | 2 (2.7) | 1.00 | 3 (1.1) | 0 (0) | 1.00 |
| Endocrine/Metabolic | 56 (21.8) | 19 (26) | 0.43 | 7 (2.7) | 0 (0) | 0.35 |
| Obesity^c^ | 135/201 (67.2) | 36/61 (59) | 0.28 | 79/252 (31.3) | 15/59 (25.4) | 0.43 |
| **Clinical** **Characteristics** | | | | | | |
| Maximum PELOD-2, median (IQR) | 1 [0, 3] | 2 [0, 6] | **<0.001** | 3 [2, 4] | 2 [2, 5] | 0.73 |
| Organ systems involved, median (IQR) | 3 [2, 4] | 3 [2, 4] | 0.43 | 5 [4, 5] | 4 [4, 5] | **0.04** |
| Intensive Care Unit admission, n (%) | 174 (67.7) | 59 (80.8) | **0.03** | 214 (82) | 48 (78.7) | 0.58 |
| Mechanical ventilation, n (%)^d^ | 111 (43.2) | 41 (56.2) | 0.06 | 69 (26.4) | 16 (26.2) | 1.00 |
| Length of mechanical ventilation (days), median (IQR)^d^ | 0 [0, 2] | 0 [0, 0] | 0.15 | 0 [0, 2] | 0 [0, 3] | 0.37 |
| Cardiovascular dysfunction, n (%) | 49 (19.1) | 22 (30.1) | 0.05 | 191 (73.2) | 48 (78.7) | 0.42 |
| Extracorporeal membrane oxygenation, n (%) | 9 (3.5) | 4 (5.5) | 0.49 | 9 (3.4) | 0 (0) | 0.22 |
| Duration of Intensive Care Unit stay (days), median (IQR) | 5 [3, 11] | 6 [3, 16.5] | **0.01** | 3 [2, 5] | 3 [2, 5] | 0.39 |
| Duration of Hospitalization (days), median (IQR) | 6 [3, 13] | 8 [4, 21] | **0.02** | 6 [5, 8] | 5 [4, 7] | **0.02** |

^a^Patients with follow-up were those with a known outcome at 2-4 months including outpatient follow-up data collected, readmitted to the hospital at 2-4 month follow-up, or without 2-4 month outcome data but returned to baseline at 1-month follow-up time point.

^b^Pre-existing condition categories were not mutually exclusive.

^c^Obesity was defined by national reference standards for body mass index if age > 2 years and was considered separately from other pre-existing conditions (16).

^d^Mechanical ventilation duration includes invasive and non-invasive modes of support.

IQR: interquartile range; PELOD-2: Pediatric Logistic Organ Dysfunction-2 score.

**Table S5** – Comparison of Acute COVID-19 and MIS-C Patient and Clinical Characteristics by Strain-Predominant Time Period

| **Characteristic, n (%)** | **Acute COVID-19** | | | **MIS-C** | | |
| --- | --- | --- | --- | --- | --- | --- |
|  | **Pre-Delta** | **Delta** | **Omicron** | **Pre-Delta** | **Delta** | **Omicron** |
| n (%) | 127/232 (54.7) | 91/232 (39.2) | 14/232 (6.0) | 170/241 (70.5) | 49/241 (20.3) | 22/241 (9.1) |
| Age Group (years), n (%) | | | | | | |
| <2 | 30 (23.6) | 13 (14.3) | 7 (50) | 8 (4.7) | 0 (0) | 2 (9.1) |
| 2-<5 | 10 (7.9) | 4 (4.4) | 2 (14.3) | 22 (12.9) | 4 (8.2) | 4 (18.2) |
| 5-<13 | 25 (19.7) | 21 (23.1) | 0 (0) | 76 (44.7) | 32 (65.3) | 15 (68.2) |
| 13-<21 | 62 (48.8) | 53 (58.2) | 5 (35.7) | 64 (37.6) | 13 (26.5) | 1 (4.5) |
| Male sex, n (%) | 65 (51.2) | 37 (40.7) | 9 (64.3) | 101 (59.4) | 31 (63.3) | 13 (59.1) |
| **Race and Ethnicity, n (%)** | | | | | | |
| White, non-Hispanic | 45 (35.4) | 47 (51.6) | 10 (71.4) | 54 (31.8) | 16 (32.7) | 10 (45.5) |
| Black, non-Hispanic | 29 (22.8) | 18 (19.8) | 0 (0) | 62 (36.5) | 17 (34.7) | 5 (22.7) |
| Hispanic or Latino | 42 (33.1) | 20 (22) | 3 (21.4) | 39 (22.9) | 10 (20.4) | 4 (18.2) |
| Multiple/Other, non-Hispanic | 11 (8.7) | 4 (4.4) | 1 (7.1) | 12 (7.1) | 5 (10.2) | 0 (0) |
| Unknown | 0 (0) | 2 (2.2) | 0 (0) | 3 (1.8) | 1 (2) | 3 (13.6) |
| **Social Determinants of Health** | | | | | | |
| Public Insurance, n (%) | 82 (64.6) | 57 (62.6) | 5 (35.7) | 95 (55.9) | 23 (46.9) | 12 (54.5) |
| Private Insurance, n (%) | 42 (33.1) | 32 (35.2) | 9 (64.3) | 67 (39.4) | 25 (51) | 9 (40.9) |
| Unknown Insurance, n (%) | 3 (2.4) | 2 (2.2) | 0 (0) | 8 (4.7) | 1 (2) | 1 (4.5) |
| Social Vulnerability Index (SVI), median (IQR) | 0.69 [0.45, 0.86] | 0.67 [0.38, 0.83] | 0.4 [0.28, 0.58] | 0.52 [0.29, 0.81] | 0.64 [0.23, 0.86] | 0.59 [0.25, 0.84] |
| Lowest 3^rd^ SVI, n (%) | 21 (16.5) | 18 (19.8) | 6 (42.9) | 53 (31.2) | 16 (32.7) | 8 (36.4) |
| Middle 3^rd^ SVI, n (%) | 38 (29.9) | 28 (30.8) | 7 (50) | 48 (28.2) | 10 (20.4) | 5 (22.7) |
| Highest 3^rd^ SVI, n (%) | 68 (53.5) | 45 (49.5) | 1 (7.1) | 69 (40.6) | 23 (46.9) | 9 (40.9) |
| **Underlying Conditions, n (%)** | | | | | | |
| Previously Healthy | 53 (41.7) | 42 (46.2) | 6 (42.9) | 138 (81.2) | 41 (83.7) | 18 (81.8) |
| Pre-existing Respiratory Condition | 39 (30.7) | 31 (34.1) | 4 (28.6) | 21 (12.4) | 5 (10.2) | 4 (18.2) |
| Isolated asthma or reactive airways disease | 18 (14.2) | 20 (22) | 2 (14.3) | 19 (11.2) | 4 (8.2) | 4 (18.2) |
| Pre-existing non-respiratory condition | 66 (52) | 31 (34.1) | 5 (35.7) | 14 (8.2) | 4 (8.2) | 0 (0) |
| Cardiovascular | 12 (9.4) | 7 (7.7) | 1 (7.1) | 3 (1.8) | 0 (0) | 0 (0) |
| Neurologic/Neuromuscular | 28 (22) | 15 (16.5) | 4 (28.6) | 4 (2.4) | 1 (2) | 0 (0) |
| Immunocompromised | 7 (5.5) | 1 (1.1) | 1 (7.1) | 1 (0.6) | 0 (0) | 0 (0) |
| Gastrointestinal/Hepatic | 32 (25.2) | 11 (12.1) | 0 (0) | 3 (1.8) | 1 (2) | 0 (0) |
| Hematologic | 13 (10.2) | 3 (3.3) | 0 (0) | 2 (1.2) | 2 (4.1) | 0 (0) |
| Renal/Urologic | 6 (4.7) | 3 (3.3) | 0 (0) | 2 (1.2) | 0 (0) | 0 (0) |
| Endocrine/Metabolic | 31 (24.4) | 15 (16.5) | 3 (21.4) | 7 (4.1) | 0 (0) | 0 (0) |
| Obesity | 41/97 (42.3) | 53/78 (67.9) | 2/7 (28.6) | 53/162 (32.7) | 13/49 (26.5) | 6/20 (30) |
| **Clinical** **Characteristics** | | | | | | |
| Maximum PELOD-2, median (IQR) | 0 [0, 2.5] | 2 [0, 3] | 5 [0.5, 6.75] | 3 [2, 5] | 2 [2, 4] | 2.5 [2, 4] |
| Organ systems involved, median (IQR) | 2 [1, 3] | 3 [2, 4] | 4 [2, 5] | 5 [4, 6] | 5 [4, 5] | 4 [4, 5] |
| Intensive Care Unit admission, n (%) | 67 (52.8) | 70 (76.9) | 14 (100) | 142 (83.5) | 38 (77.6) | 18 (81.8) |
| Mechanical ventilation, n (%)^a^ | 34 (26.8) | 50 (54.9) | 13 (92.9) | 48 (28.2) | 11 (22.4) | 6 (27.3) |
| Length of mechanical ventilation (days), median (IQR)^a^ | 0 [0, 0.5] | 0 [0, 0] | 0 [0, 1.5] | 0 [0, 1] | 0 [0, 3] | 0 [0, 4.75] |
| Cardiovascular dysfunction, n (%) | 22 (17.3) | 17 (18.7) | 6 (42.9) | 125 (73.5) | 36 (73.5) | 17 (77.3) |
| Extracorporeal membrane oxygenation, n (%) | 3 (2.4) | 4 (4.4) | 1 (7.1) | 8 (4.7) | 0 (0) | 1 (4.5) |
| Duration of Intensive Care Unit stay (days), median (IQR) | 4 [2, 11] | 6 [3.25, 10.75] | 5.5 [3, 15] | 4 [2, 5] | 2 [1.25, 5] | 2.5 [1, 7.25] |
| Duration of Hospitalization (days), median (IQR) | 4 [2, 10] | 7 [5, 14] | 13 [5.25, 22.75] | 6 [5, 9] | 5 [4, 8] | 5 [4, 8] |
| Treatment with systemic steroids | 51 (40.2) | 74 (81.3) | 14 (100) | 161 (94.7) | 43 (87.8) | 20 (90.9) |
| **Viral Co-infections, n (%)** | | | | | | |
| Any Viral Co-infection | 4 (3.1) | 8 (8.8) | 6 (42.9) | 8 (4.7) | 7 (14.3) | 2 (9.1) |
| Influenza | 0 (0) | 0 (0) | 0 (0) | 1 (0.6) | 0 (0) | 0 (0) |
| Respiratory Syncytial Virus | 0 (0) | 4 (4.4) | 3 (21.4) | 0 (0) | 0 (0) | 0 (0) |
| Parainfluenza | 1 (0.8) | 1 (1.1) | 0 (0) | 0 (0) | 0 (0) | 0 (0) |
| Adenovirus | 0 (0) | 0 (0) | 0 (0) | 0 (0) | 0 (0) | 1 (4.5) |
| Rhinovirus/Enterovirus | 2 (1.6) | 2 (2.2) | 5 (35.7) | 8 (4.7) | 6 (12.2) | 1 (4.5) |
| HMPV | 1 (0.8) | 1 (1.1) | 0 (0) | 0 (0) | 1 (2.0) | 0 (0) |
| **Outcomes, n (%)** | | | | | | |
| Persistent Symptoms | 31 (24.4) | 33 (36.3) | 7 (50) | 34 (20) | 16 (32.7) | 6 (27.3) |
| Activity Impairment | 20 (15.7) | 26 (28.6) | 4 (28.6) | 37 (21.8) | 18 (36.7) | 3 (13.6) |
| Persistent Symptoms or Activity Impairment | 37 (29.1) | 42 (46.2) | 8 (57.1) | 52 (30.6) | 23 (46.9) | 7 (31.8) |

^a^Mechanical ventilation duration includes invasive and non-invasive modes of support.

IQR: interquartile range; PELOD-2: Pediatric Logistic Organ Dysfunction-2 score.

**Figure S1 –** Outcomes of (A) Persistent Symptoms and (B) Ongoing Activity Impairment 2 to 4 Months After Hospitalization Among Patients Admitted for Acute COVID-19. Error bars represent 95% confidence intervals for the true proportion of survey respondents.

**
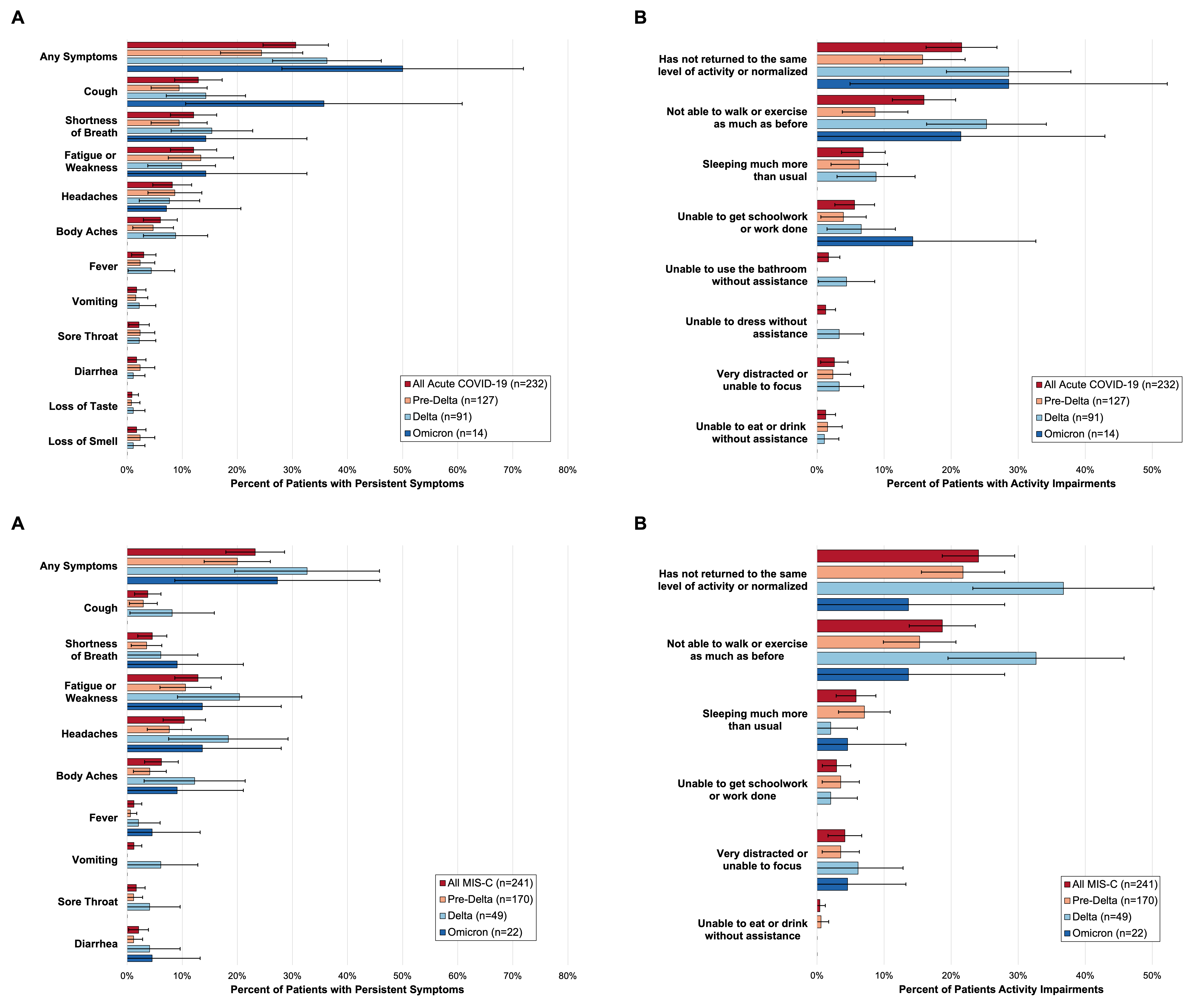
**

**
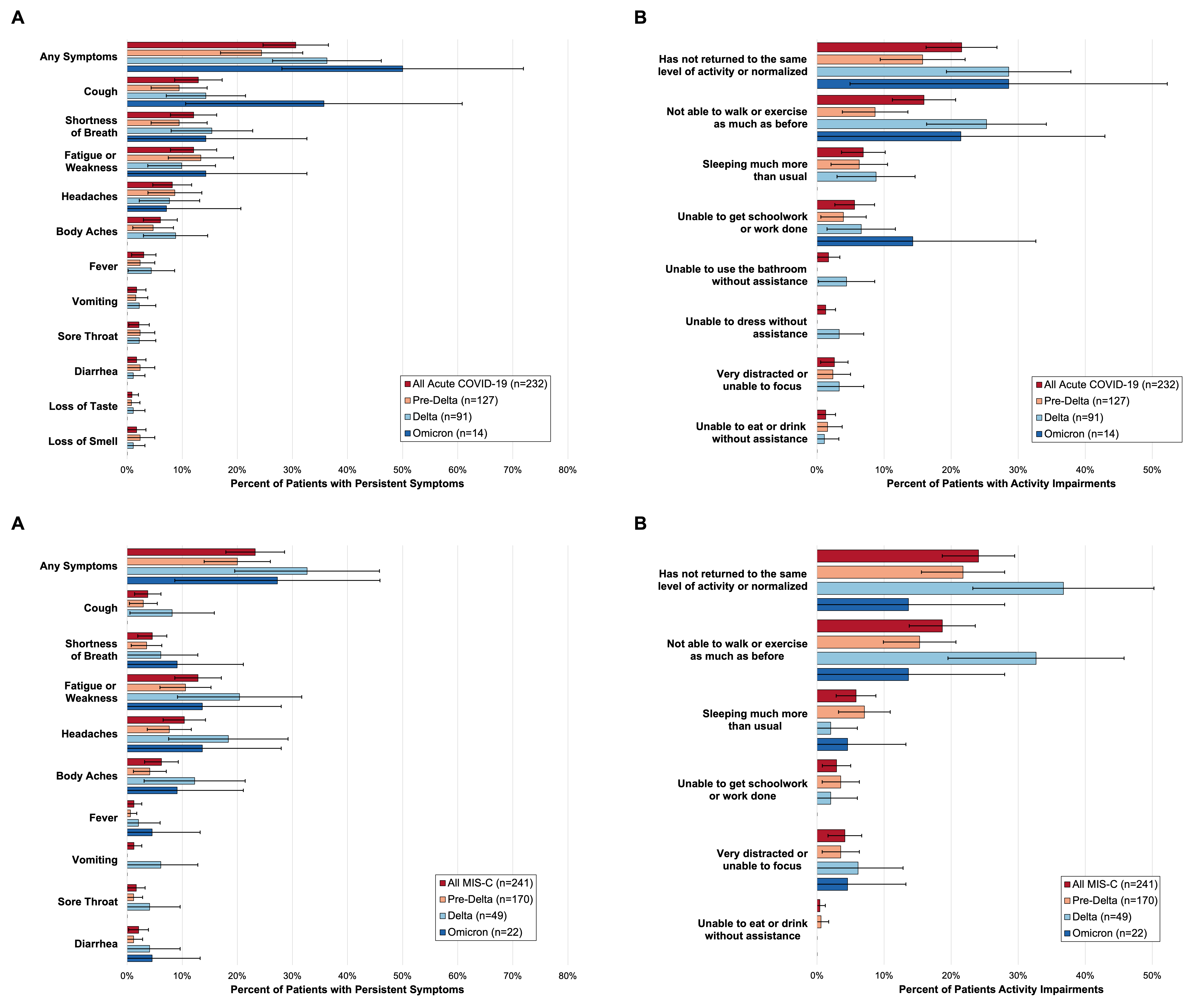
**

**Table S6** – Patient and Clinical Factors Associated With Persistent Symptoms or Activity Impairment at 2 to 4 Months After Admission in Patients with Acute COVID-19

| **Characteristic, no. (%)** | **COVID-19 Persistent Symptoms (n=71)** | **COVID-19 No Symptoms (n=161)** | **P-value** | **COVID-19 Activity Impairment (n=50)** | **COVID-19 Normal Activity (n=182)** | **P-value** |
| --- | --- | --- | --- | --- | --- | --- |
| Age Group (years), n (%) |  |  |  |  |  |  |
| <2 | 12 (16.9) | 38 (23.6) | **0.60^a^** | 5 (10) | 45 (24.7) | **0.03^a^** |
| 2-<5 | 6 (8.5) | 10 (6.2) |  | 2 (4) | 14 (7.7) |  |
| 5-<13 | 16 (22.5) | 30 (18.6) |  | 8 (16) | 38 (20.9) |  |
| 13-<21 | 37 (52.1) | 83 (51.6) |  | 35 (70) | 85 (46.7) |  |
| Male sex | 32 (45.1) | 79 (49.1) | **0.67^a^** | 25 (50) | 86 (47.3) | **0.75^a^** |
| **Race and Ethnicity, n (%)** | | | | | | |
| White, non-Hispanic | 43 (60.6) | 59 (36.6) | 0.001 | 27 (54) | 75 (41.2) | 0.30 |
| Black, non-Hispanic | 6 (8.5) | 41 (25.5) |  | 7 (14) | 40 (22) |  |
| Hispanic or Latino | 15 (21.1) | 50 (31.1) |  | 11 (22) | 54 (29.7) |  |
| Multiple/Other, non-Hispanic | 6 (8.5) | 10 (6.2) |  | 5 (10) | 11 (6.0) |  |
| Unknown | 1 (1.4) | 1 (0.6) |  | 0 (0) | 2 (1.1) |  |
| **Social Determinants of Health** | | | | | | |
| Public Insurance, n (%) | 39 (54.9) | 105 (65.2) | 0.14 | 30 (60) | 114 (62.6) | 0.14 |
| Private Insurance, n (%) | 29 (40.8) | 54 (33.5) |  | 17 (34) | 66 (36.3) |  |
| Unknown Insurance, n (%) | 3 (4.2) | 2 (1.2) |  | 3 (6) | 2 (1.1) |  |
| Social Vulnerability Index Category, n (%) |  |  |  |  |  |  |
| Lowest 3^rd^ | 15 (21.1) | 30 (18.6) | **0.001** | 10 (20) | 35 (19.2) | **0.28** |
| Middle 3^rd^ | 33 (46.5) | 40 (24.8) |  | 20 (40) | 53 (29.1) |  |
| Highest 3^rd^ | 23 (32.4) | 91 (56.5) |  | 20 (40) | 94 (51.6) |  |
| **Underlying Conditions, n (%)** | | | | | | |
| Previously Healthy | 24 (33.8) | 77 (47.8) | 0.06 | 16 (32) | 85 (46.7) | 0.08 |
| Pre-existing Respiratory Condition | 27 (38) | 47 (29.2) | **0.22** | 19 (38) | 55 (30.2) | 0.31 |
| Isolated asthma or reactive airways disease | 12 (16.9) | 28 (17.4) | 1.00 | 13 (26) | 27 (14.8) | NA |
| Pre-existing non-respiratory condition | 38 (53.5) | 64 (39.8) | **0.06** | 24 (48) | 78 (42.9) | 0.52 |
| Cardiovascular | 7 (9.9) | 13 (8.1) | NA | 5 (10) | 15 (8.2) | NA |
| Neurologic/Neuromuscular | 22 (31) | 25 (15.5) | NA | 12 (24) | 35 (19.2) | NA |
| Immunocompromised | 5 (7) | 4 (2.5) | NA | 4 (8) | 5 (2.7) | NA |
| Gastrointestinal/Hepatic | 21 (29.6) | 22 (13.7) | NA | 12 (24) | 31 (17) | NA |
| Hematologic | 4 (5.6) | 12 (7.5) | NA | 2 (4) | 14 (7.7) | NA |
| Renal/Urologic | 4 (5.6) | 5 (3.1) | NA | 2 (4) | 7 (3.8) | NA |
| Endocrine/Metabolic | 19 (26.8) | 30 (18.6) | NA | 13 (26) | 36 (19.8) | NA |
| Obesity | 32/59 (54.2) | 64/123 (52.0) | 0.87 | 33/45 (73.3) | 63/137 (46.0) | **0.002** |
| **Clinical Characteristics** | | | | | | |
| Maximum PELOD-2, median (IQR) | 2 [0, 5] | 0 [0, 2] | **<0.001** | 2 [0, 5] | 1 [0, 2] | **0.009** |
| Organ systems involved, median (IQR) | 3 [2, 4.5] | 2 [2, 3] | **<0.001** | 3.5 [2, 5] | 2 [2, 3] | **<0.001** |
| Intensive Care Unit admission, n (%) | 52 (73.2) | 99 (61.5) | 0.10 | 42 (84) | 109 (59.9) | 0.001 |
| Mechanical ventilation, n (%)^b^ | 44 (62) | 53 (32.9) | **<0.001** | 32 (64) | 65 (35.7) | **<0.001** |
| Length of Mechanical Ventilation (days), median (IQR)^b^ | 0 [0, 1] | 0 [0, 1] | 0.82 | 0 [0, 1] | 0 [0, 1] | 0.50 |
| Cardiovascular dysfunction, n (%) | 23 (32.4) | 22 (13.7) | **0.002** | 19 (38) | 26 (14.3) | **<0.001** |
| Extracorporeal membrane oxygenation, n (%) | 6 (8.5) | 2 (1.2) | 0.01 | 5 (10) | 3 (1.6) | 0.01 |
| **Clinical Outcomes** |  |  |  |  |  |  |
| Duration of Intensive Care Unit stay (days), median (IQR) | 6 [4, 20] | 5 [2, 9.5] | 0.02 | 7 [4, 20] | 5 [2, 9] | 0.005 |
| Duration of Hospitalization (days), median (IQR) | 8 [4.5, 18.5] | 5 [2, 11] | 0.002 | 10 [6, 22.75] | 5 [3, 10] | <0.001 |

Bolded p-values were those variables that were included in the multivariable model based on *a priori* designation and p-value < 0.3.

^a^Age category and sex included in every multivariable model.

^b^Mechanical ventilation duration includes invasive and non-invasive modes of support for all patients in the cohort.

IQR: interquartile range; PELOD-2: Pediatric Logistic Organ Dysfunction-2 score.

**Figure S2** – Mixed Effects Multivariable Models Evaluating Factors Associated with A) Persistent Symptoms or B) Activity Impairments in Patients with Acute COVID-19 Including Variable for Strain-Predominant Time Period

**A)**

**
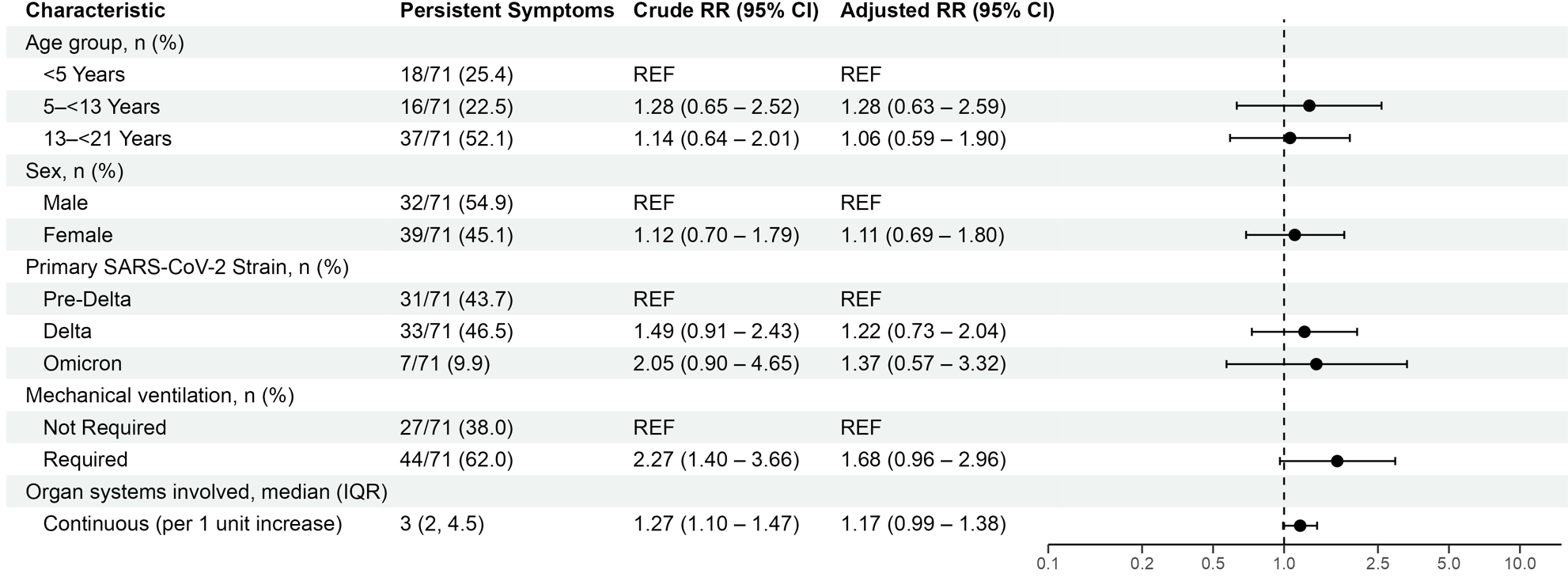
**

Variables considered for inclusion in model included: age category, sex, primary SARS-CoV-2 strain, SVI category, pre-existing respiratory condition, pre-existing non-respiratory condition, maximum PELOD-2 score, organ systems involved, mechanical ventilation, and cardiovascular dysfunction.

CI = confidence interval; RR = risk ratio; n = number; IQR = interquartile range

**B)**


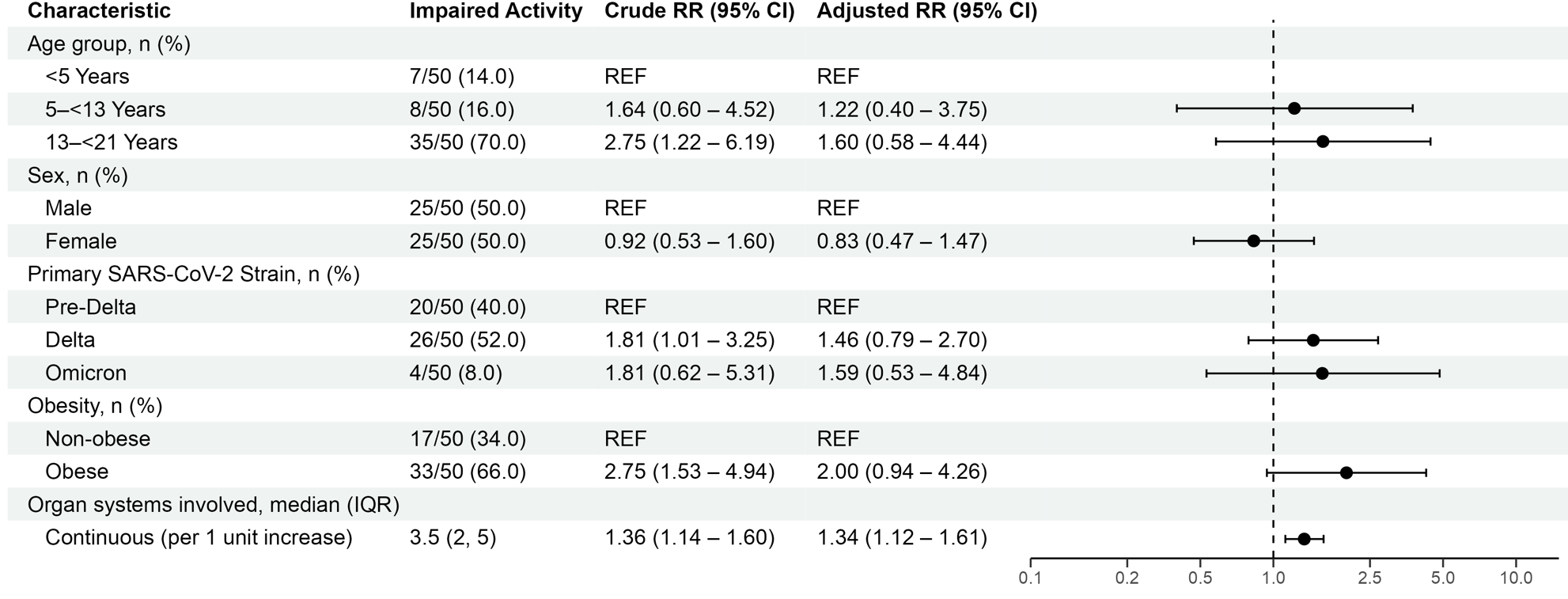


Variables considered for inclusion in models were age category, sex, SVI category, pre-existing respiratory condition, non-respiratory pre-existing condition, obesity, maximum PELOD-2 score, receipt of mechanical ventilation, duration of mechanical ventilation, cardiovascular dysfunction, and organ systems involved. Patients younger than 2-years-old were considered non-obese.

CI = confidence interval; RR = risk ratio; n = number; IQR = interquartile range

**Table S7** – Patient and Clinical Factors Associated With Persistent Symptoms or Activity Impairment at 2 to 4 Months After Admission in Patients with Acute COVID-19 Including Patients Missing 2-4-Month Follow-up But Normalized by 1 Month (sensitivity analysis)

| **Characteristic** | **COVID-19 Persistent Symptoms (n=71)** | **COVID-19 No Symptoms (n=180)** | **P-value** | **COVID-19 Activity Impairment (n=50)** | **COVID-19 Normal Activity (n=201)** | **P-value** |
| --- | --- | --- | --- | --- | --- | --- |
| Age Group (years), n (%) | | | | | | |
| <2 | 12 (16.9) | 43 (23.9) | **0.57^a^** | 5 (10) | 50 (24.9) | **0.02^a^** |
| 2-<5 | 6 (8.5) | 11 (6.1) |  | 2 (4) | 15 (7.5) |  |
| 5-<13 | 16 (22.5) | 34 (18.9) |  | 8 (16) | 42 (20.9) |  |
| 13-<21 | 37 (52.1) | 92 (51.1) |  | 35 (70) | 94 (46.8) |  |
| Male sex, n (%) | 32 (45.1) | 86 (47.8) | **0.78^a^** | 25 (50) | 93 (46.3) | **0.64^a^** |
| Primary SARS-CoV-2 Strain, n (%) | | | | | | |
| Pre-Delta | 31 (43.7) | 104 (57.8) | **0.11^a^** | 20 (40) | 115 (57.2) | **0.09^a^** |
| Delta | 33 (46.5) | 66 (36.7) |  | 26 (52) | 73 (36.3) |  |
| Omicron | 7 (9.9) | 10 (5.6) |  | 4 (8) | 13 (6.5) |  |
| Social Vulnerability Index Category, n (%) | | | | | | |
| Lowest 3^rd^ | 15 (21.1) | 34 (18.9) | **0.001** | 10 (20) | 39 (19.4) | **0.24** |
| Middle 3^rd^ | 33 (46.5) | 45 (25.0) |  | 20 (40) | 58 (28.9) |  |
| Highest 3^rd^ | 23 (32.4) | 101 (56.1) |  | 20 (40) | 104 (51.7) |  |
| Pre-existing Respiratory Condition, n (%) | 27 (38) | 52 (28.9) | **0.18** | 19 (38) | 60 (29.9) | 0.31 |
| Pre-existing Non-Respiratory Condition, n (%) | 38 (53.5) | 77 (42.8) | **0.16** | 24 (48) | 91 (45.3) | 0.75 |
| Obesity, n (%) | 32/59 (54.2) | 71/137 (51.8) | 0.88 | 33/45 (73.3) | 70/151 (46.4) | **0.002** |
| Maximum PELOD-2, median (IQR) | 2 [0, 5] | 0 [0, 2] | **<0.001** | 2 [0, 5] | 1 [0, 3] | **0.001** |
| Organ systems involved, median (IQR) | 3 [2, 4.5] | 2 [2, 3] | **0.001** | 3.5 [2, 5] | 2 [2, 3] | **<0.001** |
| Mechanical ventilation, n (%)^b^ | 44 (62) | 61 (33.9) | **<0.001** | 32 (64) | 73 (36.3) | **<0.001** |
| Length of mechanical ventilation (days), median (IQR)^b^ | 0 [0, 1] | 0 [0, 0] | **0.07** | 0 [0, 1] | 0 [0, 0] | 0.74 |
| Cardiovascular dysfunction, n (%) | 23 (32.4) | 24 (13.3) | **0.001** | 19 (38) | 28 (13.9) | **<0.001** |

Bolded p-values were those variables that were included in the multivariable model based on *a priori* designation and p-value <0.3.

^a^Age category, sex, and primary SARS-CoV-2 strain included in every multivariable model.

^b^Mechanical ventilation duration includes invasive and non-invasive modes of support for all patients in the cohort.

IQR: interquartile range; PELOD-2: Pediatric Logistic Organ Dysfunction-2 score.

**Table S8 –** Mixed Effects Multivariable Models Evaluating Factors Associated with Persistent Symptoms or Activity Impairments in Patients with Acute COVID-19 Including Variable for Strain-Predominant Time Period and Patients without 2-4-Month Follow-Up Data but Normalized at 1 Month (sensitivity analysis)

| **Characteristic^a^** | **Persistent Symptoms** | **Resolved Symptoms** | **Crude RR (95% CI)** | **Adjusted RR (95% CI)** |  |
| --- | --- | --- | --- | --- | --- |
| Age group, n (%) | | | | | |
| <5 Years | 18/71 (25.4) | 54/180 (30.0) | Reference | Reference |  |
| 5–<13 Years | 16/71 (22.5) | 34/180 (18.9) | 1.30 (0.66, 2.56) | 1.25 (0.62, 2.50) |  |
| 13–<21 Years | 37/71 (52.1) | 92/180 (51.1) | 1.16 (0.66, 2.05) | 1.08 (0.60, 1.94) |  |
| Sex, n (%) | | | | | |
| Male | 32/71 (54.9) | 86/180 (47.8) | Reference | Reference |  |
| Female | 39/71 (45.1) | 94/180 (52.2) | 1.08 (0.68, 1.73) | 1.06 (0.65, 1.72) |  |
| Primary SARS-CoV-2 strain, n (%) |  |  |  |  |  |
| Pre-Delta | 31/71 (43.7) | 104/180 (57.8) | Reference | Reference |  |
| Delta | 33/71 (46.5) | 66/180 (36.7) | 1.45 (0.89, 2.37) | 1.21 (0.72, 2.02) |  |
| Omicron | 7/71 (9.9) | 10/180 (5.6) | 1.79 (0.79, 4.07) | 1.20 (0.50, 2.86) |  |
| Mechanical Ventilation, n (%) | | | | | |
| Not Required | 27/71 (38.0) | 119/180 (66.1) | Reference | Reference |  |
| Required | 44/71 (62.0) | 61/180 (33.9) | 2.27 (1.40, 3.66) | 1.73 (0.99, 3.03) |  |
| Organ Systems Involved, median (IQR) | | | | | |
| Continuous (per 1 unit increase) | 3 (2, 4.5) | 2 (2, 3) | 1.27 (1.09, 1.47) | 1.17 (0.99, 1.39) |  |
| **Characteristic^b^** | **Impaired Activity** | **Normal Activity** | **Crude RR (95% CI)** | **Adjusted RR (95% CI)** |  |
| Age group, n (%) | | | | | |
| <5 Years | 7/50 (14.0) | 65/201 (32.3) | Reference | Reference |  |
| 5–<13 Years | 8/50 (16.0) | 42/201 (20.9) | 1.64 (0.60, 4.52) | 1.21 (0.40, 3.72) |  |
| 13–<21 Years | 35/50 (70.0) | 94/201 (46.8) | 2.75 (1.22, 6.19) | 1.69 (0.61, 4.68) |  |
| Sex, n (%) | | | | | |
| Male | 25/50 (50.0) | 93/201 (46.3) | Reference | Reference |  |
| Female | 25/50 (50.0) | 108/201 (53.7) | 0.92 (0.53, 1.60) | 0.78 (0.44, 1.39) |  |
| Primary SARS-CoV-2 strain, n (%) |  |  |  |  |  |
| Pre-Delta | 20/50 (40.0) | 115/201 (57.2) | Reference | Reference |  |
| Delta | 26/50 (52.0) | 73/201 (36.3) | 1.81 (1.01, 3.25) | 1.46 (0.79, 2.69) |  |
| Omicron | 4/50 (8.0) | 13/201 (6.5) | 1.81 (0.62, 5.31) | 1.41 (0.47, 4.23) |  |
| Obesity, n (%) | | | | | |
| Non-obese | 17/50 (34.0) | 131/201 (65.2) | Reference | Reference |  |
| Obese | 33/50 (66.0) | 70/201 (34.8) | 2.75 (1.53, 4.94) | 1.95 (0.92, 4.12) |  |
| Organ Systems Involved, median (IQR) | | | | | |
| Continuous (per 1 unit increase) | 3.5 (2, 5) | 2 (2, 3) | 1.36 (1.14, 1.60) | **1.35 (1.12, 1.62)** |  |

CI = confidence interval; RR = risk ratio; n = number; IQR = interquartile range

^a^Variables considered for inclusion in model included age category, sex, primary SARS-CoV-2 strain, SVI category, underlying respiratory condition, underlying non-respiratory condition, maximum PELOD-2 score, organ systems involved, receipt of mechanical ventilation, length of mechanical ventilation, and cardiovascular involvement.

^b^Variables considered for inclusion in model included age category, sex, primary SARS-CoV-2 strain, SVI category, obesity, maximum PELOD-2 score, organ systems involved, receipt of mechanical ventilation, and cardiovascular involvement.

**Table S9** – Health-Related Quality of Life Prior to Illness and at Follow-up Among Patients with Acute COVID-19 and MIS-C Enrolled during the Second Year of the Study

| **Year 2 Cohort: PedsQL data collection** | **Acute COVID (n=113)** | **MIS-C (n=81)** |
| --- | --- | --- |
| Patients ≤4 years-old^a^, n (%) | 37 (32.7) | 24 (29.6) |
| Patients eligible for PedsQL data collection, n (%) | 76 (67.3) | 57 (70.4) |
| Missing pre-illness baseline, n (%) | 4 (5.3) | 4 (7.0) |
| Missing follow-up, n (%) | 2 (2.6) | 0 (0) |
| Time from hospital admission to follow-up Peds QL data collection (days), median (IQR) and range | 64 (61, 71); n=70  Range: 50–101 | 64 (60, 67); n=53  Range: 50–90 |
| **Data for patients with baseline and follow-up data collected** | | |
| n (%) | 70 (92.1) | 53 (93.0) |
| PedsQL pre-illness Total Health Summary score, median (IQR) | 88.3 (66.9, 97.8) | 94.6 (85.9, 100) |
| Follow-up PedsQL Total Health Summary score, median (IQR) | 83.2 (56.8, 97.8) | 94.6 (81.5, 98.9) |
| Change in PedsQL Total Health Summary score, median (IQR) | 0 (-12.5, 7.4) | 0 (-7.6, 5.4) |
| Change in PedsQL Physical Health Summary score, median (IQR) | -3.1 (-18.8, 2.3) | 0 (-9.4, 0) |
| Change in PedsQL Psychosocial Health Summary score, median (IQR) | 1.7 (-8.2, 11.3) | 0 (-6.7, 8.3) |
| Patients with follow-up PedsQL Total Health Summary >4.5 points below pre-illness baseline, n (%)^b^ | 25 (35.7) | 18 (34.0) |
| Patients with follow-up PedsQL Total Health Summary >18 points below pre-illness baseline, n (%)^b^ | 15 (21.4) | 6 (11.3) |
| Patients with follow-up PedsQL Physical Health Summary >6.92 points below pre-illness baseline, n (%)^b^ | 24 (34.3) | 15 (28.3) |
| Patients with follow-up PedsQL Psychosocial Health Summary score >5.49 points below pre-illness baseline, n (%)^b^ | 21 (30.0) | 16 (30.2) |
| **Patients without pre-illness PedsQL scores but with follow-up scores** | | |
| n | 4 | 4 |
| Patients with PedsQL Total Health Summary score >80, n (%)^b^ | 2/4 (50) | 2/4 (50) |

^a^Data systematically not collected for patient <4 years old.

^b^Minimally clinically important difference: 4.5 points for the PedsQL Total Health Summary score, 6.92 points for the PedsQL Physical Health Summary score, 5.49 points for the PedsQL Psychosocial Health Summary score. Severely diminished health-related quality of life delineated as 18 points below pre-illness baseline which is 4 times minimally clinically important difference. Population mean for the PedsQL Total Health Summary Score is 82.87 (child self-report) and 81.34 (parent proxy-report), therefore, total score >80 represents a normal HRQL relative to a general pediatric population(17).

**Table S10** – Patient and Clinical Factors Associated With Persistent Symptoms or Activity Impairment at 2 to 4 Months After Admission in Patients with MIS-C

| **Characteristic, no. (%)** | **MIS-C Persistent Symptoms (n=56)** | **MIS-C No Symptoms (n=185)** | **P-value** | **MIS-C Activity Impairment (n=58)** | **MIS-C Normal Activity (n=183)** | **P-value** |
| --- | --- | --- | --- | --- | --- | --- |
| Age Group, n (%) | | | | | | |
| <2 Years | 0 (0) | 10 (5.4) | **0.37^a^** | 1 (1.7) | 9 (4.9) | **0.14^a^** |
| 2-<5 Years | 7 (12.5) | 23 (12.4) |  | 4 (6.9) | 26 (14.2) |  |
| 5-<13 Years | 31 (55.4) | 92 (49.7) |  | 28 (48.3) | 95 (51.9) |  |
| 13-<21 Years | 18 (32.1) | 60 (32.4) |  | 25 (43.1) | 53 (29) |  |
| Male sex | 33 (58.9) | 112 (60.5) | **0.88^a^** | 35 (60.3) | 110 (60.1) | **1.00^a^** |
| **Race and Ethnicity** | | | | | | |
| White, non-Hispanic | 22 (39.3) | 58 (31.4) | 0.42 | 20 (34.5) | 60 (32.8) | 0.50 |
| Black, non-Hispanic | 14 (25) | 70 (37.8) |  | 16 (27.6) | 68 (32.2) |  |
| Hispanic or Latino | 13 (23.2) | 40 (21.6) |  | 14 (24.1) | 39 (21.3) |  |
| Multiple/Other, non-Hispanic | 5 (8.9) | 12 (6.5) |  | 5 (8.6) | 12 (6.6) |  |
| Unknown | 2 (3.6) | 5 (2.7) |  | 3 (5.2) | 4 (2.2) |  |
| **Social Determinants of Health** | | | | | | |
| Public Insurance | 29 (51.8) | 101 (54.6) | 0.83 | 31 (53.4) | 99 (54.1) | 0.62 |
| Private Insurance | 24 (42.9) | 77 (41.6) |  | 26 (44.8) | 75 (41) |  |
| Unknown Insurance | 3 (5.4) | 7 (3.8) |  | 1 (1.7) | 9 (4.9) |  |
| Social Vulnerability Index Category, n (%) |  |  |  |  |  |  |
| Lowest 3^rd^ | 19 (33.9) | 58 (31.4) | 0.94 | 19 (32.8) | 58 (31.7) | **0.17^a^** |
| Middle 3^rd^ | 14 (25) | 49 (26.5) |  | 10 (17.2) | 53 (29) |  |
| Highest 3^rd^ | 23 (41.1) | 78 (42.2) |  | 29 (50) | 72 (39.3) |  |
| **Underlying Conditions** | | | | | | |
| Previously Healthy | 38 (67.9) | 159 (85.9) | 0.005 | 47 (81) | 150 (82) | 0.85 |
| Pre-existing Respiratory Condition | 17 (30.4) | 13 (7) | **<0.001** | 8 (13.8) | 22 (12) | 0.82 |
| Isolated asthma or RAD | 17 (30.4) | 10 (5.4) | <0.001 | 7 (12.1) | 20 (10.9) | NA |
| Pre-existing non-respiratory condition | 4 (7.1) | 14 (7.6) | 1.00 | 4 (6.9) | 14 (7.7) | 1.00 |
| Cardiovascular | 0 (0) | 3 (1.6) | NA | 0 (0) | 3 (1.6) | NA |
| Neurologic/Neuromuscular | 2 (3.6) | 3 (1.6) | NA | 1 (1.7) | 4 (2.2) | NA |
| Immunocompromised | 0 (0) | 1 (0.5) | NA | 0 (0) | 1 (0.5) | NA |
| Gastrointestinal/Hepatic | 0 (0) | 4 (2.2) | NA | 1 (1.7) | 3 (1.6) | NA |
| Hematologic | 2 (3.6) | 2 (1.1) | NA | 1 (1.7) | 3 (1.6) | NA |
| Renal/Urologic | 0 (0) | 2 (1.1) | NA | 0 (0) | 2 (1.1) | NA |
| Endocrine/Metabolic | 0 (0) | 7 (3.8) | NA | 1 (1.7) | 6 (3.3) | NA |
| Obesity | 19/56 (33.9) | 53/175 (30.3) | 0.62 | 28/57 (49.1) | 44/174 (25.3) | **0.002** |
| **Clinical Characteristics** | | | | | | |
| Maximum PELOD-2, median (IQR) | 3 [2, 5] | 2 [1, 4] | **0.21** | 3 [2, 5] | 2 [1.5, 4] | **0.21** |
| Organ systems involved, median (IQR) | 5 [4, 6] | 5 [4, 6] | **0.25** | 5 [4.25, 6] | 5 [4, 5] | **0.003** |
| Intensive Care Unit admission, n (%) | 46 (82.1) | 152 (82.2) | 1.00 | 48 (82.8) | 150 (82) | 1.00 |
| Mechanical ventilation, n (%)^a^ | 17 (30.4) | 48 (25.9) | 0.50 | 20 (34.5) | 45 (24.6) | **0.17** |
| Length of Mechanical Ventilation (days), median (IQR)^a^ | 0 [0, 1.5] | 0 [0, 3] | 0.85 | 0 [0, 2.5] | 0 [0, 3] | 0.91 |
| Cardiovascular dysfunction, n (%) | 40 (71.4) | 138 (74.6) | 0.73 | 43 (74.1) | 135 (73.8) | 1.00 |
| Extracorporeal membrane oxygenation, n (%) | 4 (7.1) | 5 (2.7) | 0.22 | 3 (5.2) | 6 (3.3) | 0.45 |
| **Clinical Outcomes** |  |  |  |  |  |  |
| Duration of Intensive Care Unit stay (days), median (IQR) | 3.5 [2, 6] | 3 [2, 5] | 0.43 | 3.5 [2, 6.25] | 3 [2, 5] | 0.32 |
| Duration of Hospitalization (days), median (IQR) | 8 [5, 10.25] | 6 [4, 8] | 0.02 | 8 [5, 11] | 6 [4, 8] | <0.001 |

Bolded p-values were those variables that were included in the multivariable model based on *a priori* designation and p-value < 0.3.

^a^Age category and sex included in every multivariable model.

^b^Mechanical ventilation duration includes invasive and non-invasive modes of support for all patients in the cohort.

IQR: interquartile range; PELOD-2: Pediatric Logistic Organ Dysfunction-2 score.

**Table S11** – Patient and Clinical Factors Associated With Persistent Symptoms or Activity Impairment at 2 to 4 Months After Admission in Patients with MIS-C Including Patients Missing 2-4-Month Follow-up But Normalized by 1 Month (sensitivity analysis)

| **Characteristic** | **MIS-C Persistent Symptoms (n=56)** | **MIS-C No Symptoms (n=198)** | **P-value** | **MIS-C Activity Impairment (n=58)** | **MIS-C Normal Activity (n=196)** | **P-value** |
| --- | --- | --- | --- | --- | --- | --- |
| Age Group, n (%) | | | | | | |
| <2 Years | 0 (0) | 10 (5.1) | **0.40^a^** | 1 (1.7) | 9 (4.6) | **0.09^a^** |
| 2-<5 Years | 7 (12.5) | 27 (13.6) |  | 4 (6.9) | 30 (15.3) |  |
| 5-<13 Years | 31 (55.4) | 99 (50) |  | 28 (48.3) | 102 (52.0) |  |
| 13-<21 Years | 18 (32.1) | 62 (31.3) |  | 25 (43.1) | 55 (28.1) |  |
| Male sex, n (%) | 33 (58.9) | 118 (59.6) | **1.00^a^** | 35 (60.3) | 116 (59.2) | **1.00^a^** |
| Social Vulnerability Index Category, n (%) |  |  |  |  |  |  |
| Lowest 3^rd^ | 19 (33.9) | 62 (31.3) | 0.89 | 19 (32.8) | 62 (31.6) | **0.09** |
| Middle 3^rd^ | 14 (25) | 56 (28.3) |  | 10 (17.2) | 60 (30.6) |  |
| Highest 3^rd^ | 23 (41.1) | 80 (40.4) |  | 29 (50) | 74 (37.8) |  |
| Pre-existing Respiratory Condition, n (%) | 17 (30.4) | 15 (7.6) | **<0.001** | 8 (13.8) | 24 (12.2) | 0.82 |
| Pre-existing Non-Respiratory Condition, n (%) | 4 (7.1) | 15 (7.6) | 1.00 | 4 (6.9) | 15 (7.7) | 1.00 |
| Obesity, n (%) | 19/56 (33.9) | 55/188 (29.3) | 0.51 | 28/57 (49.1) | 46/187 (24.6) | **<0.001** |
| Maximum PELOD-2, median (IQR) | 3 [2, 5] | 2.5 [1, 4] | **0.21** | 3 [2, 5] | 2 [2, 4] | **0.21** |
| Organ systems involved, median (IQR) | 5 [4, 6] | 5 [4, 5] | **0.18** | 5 [4, 6] | 5 [4, 5] | **0.003** |
| Mechanical ventilation, n (%)^a^ | 17 (30.4) | 49 (24.7) | 0.39 | 20 (34.5) | 46 (23.5) | **0.12** |
| Length of mechanical ventilation (days), median (IQR)^a^ | 0 [0, 1.5] | 0 [0, 2] | 0.85 | 0 [0, 2.5] | 0 [0, 2] | 0.99 |
| Cardiovascular dysfunction, n (%) | 40 (71.4) | 146 (73.7) | 0.73 | 43 (74.1) | 143 (73.0) | 1.00 |

Bolded p-values were those variables that were included in the multivariable model based on *a priori* designation and p-value <0.3.

^a^Age category, sex, and primary SARS-CoV-2 strain included in every multivariable model.

^b^Mechanical ventilation duration includes invasive and non-invasive modes of support for all patients in the cohort.

IQR: interquartile range; PELOD-2: Pediatric Logistic Organ Dysfunction-2 score.

**Table S12 –** Mixed Effects Multivariable Models Evaluating Factors Associated with Persistent Symptoms or Activity Impairments in Patients with MIS-C Including Patients without 2-4-Month Follow-Up Data but Normalized at 1 Month (sensitivity analysis)

| **Characteristic^a^** | **Persistent Symptoms** | **Resolved Symptoms** | **Crude RR (95% CI)** | **Adjusted RR (95% CI)** |  |
| --- | --- | --- | --- | --- | --- |
| Age group, n (%) | | | | | |
| <5 Years | 7/56 (12.5) | 37/198 (18.7) | Reference | Reference |  |
| 5–<13 Years | 31/56 (55.4) | 99/198 (50.0) | 1.50 (0.66, 3.40) | 1.33 (0.58, 3.03) |  |
| 13–<21 Years | 18/56 (32.1) | 62/198 (31.3) | 1.41 (0.59, 3.39) | 1.12 (0.46, 2.75) |  |
| Sex, n (%) | | | | | |
| Male | 33/56 (58.9) | 118/198 (59.6) | Reference | Reference |  |
| Female | 23/56 (41.1) | 80/198 (40.4) | 1.02 (0.60, 1.74) | 1.03 (0.60, 1.76) |  |
| Pre-Existing Respiratory Condition, n (%) | | | | | |
| Not Present | 39/56 (69.6) | 183/198 (92.4) | Reference | Reference |  |
| Present | 17/56 (30.4) | 15/198 (7.6) | 3.02 (1.71, 5.35) | **2.98 (1.67, 5.30)** |  |
| Maximum PELOD-2 Score, median (IQR) | | | | | |
| Continuous (per 1 unit increase) | 3 (2, 5) | 2.5 (1, 4) | 1.07 (0.97, 1.19) | 1.05 (0.93, 1.19) |  |
| Organ Systems Involved, median (IQR) | | | | | |
| Continuous (per 1 unit increase) | 5 (4, 6) | 5 (4, 5) | 1.13 (0.91, 1.41) | 1.06 (0.82, 1.37) |  |
| **Characteristic^b^** | **Impaired Activity** | **Normal Activity** | **Crude RR (95% CI)** | **Adjusted RR (95% CI)** |  |
| Age group, n (%) | | | | | |
| <5 Years | 5/58 (8.6) | 39/196 (19.9) | Reference | Reference |  |
| 5–<13 Years | 28/58 (48.3) | 102/196 (52.0) | 1.90 (0.73, 4.92) | 1.76 (0.68, 4.56) |  |
| 13–<21 Years | 25/58 (43.1) | 55/196 (28.1) | 2.75 (1.05, 7.22) | 2.32 (0.88, 6.11) |  |
| Sex, n (%) | | | | | |
| Male | 35/58 (60.3) | 116/196 (59.2) | Reference | Reference |  |
| Female | 23/58 (39.7) | 80/196 (40.8) | 0.97 (0.57, 1.64) | 1.00 (0.59, 1.69) |  |
| Obesity, n (%) | | | | | |
| Non-obese | 30/58 (51.7) | 150/196 (76.5) | Reference | Reference |  |
| Obese | 28/58 (48.3) | 46/196 (23.5) | 2.27 (1.35, 3.81) | **1.88 (1.11, 3.19)** |  |
| Organ Systems Involved), median (IQR) | | | | | |
| Continuous (per 1 unit increase) | 5 (4, 6) | 5 (4, 5) | 1.35 (1.08, 1.69) | **1.27 (1.01, 1.59)** |  |

CI = confidence interval; RR = risk ratio; n = number; IQR = interquartile range

^a^Variables considered for inclusion in model included age category, sex, underlying respiratory condition, maximum PELOD-2 score, organ systems involved, and receipt of mechanical ventilation.

^b^Variables considered for inclusion in model included age category, sex, SVI category, obesity, maximum PELOD-2 score, organ systems involved, and receipt of mechanical ventilation.

References:

1. Asadi-Pooya AA, Nemati H, Shahisavandi M *et al*: Long COVID in children and adolescents. *World J Pediatr* 2021; 17:495-499.

2. Osmanov IM, Spiridonova E, Bobkova P *et al*: Risk factors for long covid in previously hospitalised children using the ISARIC Global follow-up protocol: A prospective cohort study. *Eur Respir J* 2021.

3. Rao S, Lee GM, Razzaghi H *et al*: Clinical Features and Burden of Postacute Sequelae of SARS-CoV-2 Infection in Children and Adolescents. *JAMA Pediatr* 2022; 176:1000-1009.

4. Baptista de Lima J, Salazar L, Fernandes A *et al*: Long COVID in Children and Adolescents: A Retrospective Study in a Pediatric Cohort. *Pediatr Infect Dis J* 2023; 42:e109-e111.

5. Pazukhina E, Andreeva M, Spiridonova E *et al*: Prevalence and risk factors of post-COVID-19 condition in adults and children at 6 and 12 months after hospital discharge: a prospective, cohort study in Moscow (StopCOVID). *BMC Med* 2022; 20:244.

6. Valenzuela G, Alarcón-Andrade G, Schulze-Schiapacasse C *et al*: Short-term complications and post-acute sequelae in hospitalized paediatric patients with COVID-19 and obesity: A multicenter cohort study. *Pediatr Obes* 2023; 18:e12980.

7. Penner J, Abdel-Mannan O, Grant K *et al*: 6-month multidisciplinary follow-up and outcomes of patients with paediatric inflammatory multisystem syndrome (PIMS-TS) at a UK tertiary paediatric hospital: a retrospective cohort study. *Lancet Child Adolesc Health* 2021; 5:473-482.

8. Kahn R, Berg S, Berntson L *et al*: Population-based study of multisystem inflammatory syndrome associated with COVID-19 found that 36% of children had persistent symptoms. *Acta Paediatr* 2022; 111:354-362.

9. Awasthi P, Kumar V, Naganur S *et al*: Multisystem Inflammatory Syndrome in Children: Follow-Up of a Cohort from North India. *Am J Trop Med Hyg* 2022; 106:1108-1112.

10. Sezer M, Çelikel E, Tekin ZE *et al*: Multisystem inflammatory syndrome in children: clinical presentation, management, and short- and long-term outcomes. *Clin Rheumatol* 2022; 41:3807-3816.

11. Son MBF, Berbert L, Young C *et al*: Postdischarge Glucocorticoid Use and Clinical Outcomes of Multisystem Inflammatory Syndrome in Children. *JAMA Netw Open* 2022; 5:e2241622.

12. Otten MH, Buysse CMP, Buddingh EP *et al*: Neurocognitive, Psychosocial, and Quality of Life Outcomes After Multisystem Inflammatory Syndrome in Children Admitted to the PICU. *Pediatr Crit Care Med* 2023; 24:289-300.

13. Flanagan BE, Hallisey EJ, Adams E, Lavery A: Measuring Community Vulnerability to Natural and Anthropogenic Hazards: The Centers for Disease Control and Prevention's Social Vulnerability Index. *J Environ Health* 2018; 80:34-36.

14. Defining childhood weight status, BMI for children and teens Available at: [<https://www.cdc.gov/obesity/childhood/defining.html>]. Accessed February 23.

15. Leteurtre S, Duhamel A, Salleron J *et al*: PELOD-2: an update of the PEdiatric logistic organ dysfunction score. *Crit Care Med* 2013; 41:1761-1773.

16. Division of Nutrition, Physical Activity, and Obesity, National Center for Chronic Disease Prevention and Health Promotion: Defining Childhood Weight Status: BMI for Children and Teens Available at: [<https://www.cdc.gov/obesity/childhood/defining.html>]. Accessed September 2, 2021.

17. Varni JW, Burwinkle TM, Seid M, Skarr D: The PedsQL 4.0 as a pediatric population health measure: feasibility, reliability, and validity. *Ambul Pediatr* 2003; 3:329-341.
